# Complexity of Secure Sets

**DOI:** 10.1007/s00453-017-0358-5

**Published:** 2017-08-14

**Authors:** Bernhard Bliem, Stefan Woltran

**Affiliations:** Institute of Information Systems 184/2, TU Wien, Favoritenstrasse 9-11, 1040 Vienna, Austria

**Keywords:** Secure set, Complexity analysis, Parameterized complexity, Treewidth, Parameterized algorithms

## Abstract

A secure set *S* in a graph is defined as a set of vertices such that for any $X\subseteq S$ the majority of vertices in the neighborhood of *X* belongs to *S*. It is known that deciding whether a set *S* is secure in a graph is $\mathrm {\text {co-}NP}$-complete. However, it is still open how this result contributes to the actual complexity of deciding whether for a given graph *G* and integer *k*, a non-empty secure set for *G* of size at most *k* exists. In this work, we pinpoint the complexity of this problem by showing that it is $\mathrm {\Sigma ^P_2}$-complete. Furthermore, the problem has so far not been subject to a parameterized complexity analysis that considers structural parameters. In the present work, we prove that the problem is $\mathrm {W[1]}$-hard when parameterized by treewidth. This is surprising since the problem is known to be FPT when parameterized by solution size and “subset problems” that satisfy this property usually tend to be FPT for bounded treewidth as well. Finally, we give an upper bound by showing membership in $\mathrm {XP}$, and we provide a positive result in the form of an FPT algorithm for checking whether a given set is secure on graphs of bounded treewidth.

## Introduction

The objective of many problems that can be modeled as graphs is finding a group of vertices that together satisfy some property. In this respect, one of the concepts that has been quite extensively studied [31] is the notion of a defensive alliance [20], which is a set of vertices such that for each element *v* at least half of its neighbors are also in the alliance. The name “defensive alliance” stems from the intuition that the neighbors of such an element *v* that are also in the alliance can help out in case *v* is attacked by its other neighbors. Notions like this can be applied to finding groups of nations, companies or individuals that depend on each other, but also to more abstract situations like finding groups of websites that form communities [18].

In this work, we are looking at a natural generalization of defensive alliances called *secure sets*, which have been introduced by Brigham et al. [11]. While defensive alliances make sure that each element of an alliance can defend itself against attacks from its neighbors, they do not account for attacks on multiple vertices at the same time. To this end, we can employ a stronger concept: A secure set of a graph *G* is a subset *S* of the vertices of *G* such that for each $X\subseteq S$, the number of vertices in $N[X]\cap S$ is not less than the number of vertices in $N[X]{\setminus } S$. Here *N*[*X*] denotes the closed neighborhood of *X* in *G*, i.e., *X* together with all vertices adjacent to *X*. The Secure Set problem can now be stated as follows: Given a graph *G* and an integer *k*, does there exists a secure set *S* of *G* such that $1\le {|S |} \le k$?

It is known that deciding whether a *given* set *S* is secure in a graph is $\mathrm {\text {co-}NP}$-complete [21], so it would not be surprising if *finding* (non-trivial) secure sets is also a very hard problem. Unfortunately, the exact complexity of this problem has so far remained unresolved. This is an unsatisfactory state of affairs because it leaves the possibility open that existing approaches for solving the problem (e.g., [1]) are suboptimal in that they employ unnecessarily powerful programming techniques. Hence we require a precise complexity-theoretic classification of the problem.

Due to its high complexity, it makes sense to look at the parameterized complexity [13, 16, 19, 26] of the problem and to study if Secure Set becomes tractable under the assumption that certain parameters of the problem instances are small. For some parameters, this may be a reasonable assumption in practice. For instance, it has been shown that Secure Set can be solved in linear time if the solution size is bounded by a constant [17]. If we are only interested in small secure sets, the resulting algorithm is therefore a good choice.

However, we often cannot make the assumption that the solutions are small. In such cases, it is a common strategy to consider structural parameters instead, which measure in a certain way how complex the graph underlying a problem instance is. One of the most studied structural parameters is treewidth [5, 7, 27], which indicates how close a graph is to being a tree. Treewidth is an attractive parameter because many hard problems become tractable on instances of bounded treewidth, and in several practical applications it has been observed that the considered problem instances exhibit small treewidth [5, 24, 30]. In [22] it has been shown that a certain variant of Secure Set becomes easy on trees, but the complexity of Secure Set parameterized by treewidth is listed as an open problem in that work and has so far remained unresolved.

The first main contribution of our paper is to show that Secure Set is $\mathrm {\Sigma ^P_2}$-complete. Unlike the existing $\mathrm {\text {co-}NP}$-hardness proof [21], which uses a (quite involved) reduction from Dominating Set, we base our proof on a reduction from a problem in the area of logic. To be specific, we first show that the canonical $\mathrm {\Sigma ^P_2}$-complete problem $\textsc {Qsat}_\textsc {2}$ can be reduced to a variant of Secure Set, where vertices can be forced to be in or out of every solution, and pairs of vertices can be specified to indicate that every solution must contain exactly one element of each such pair. In order to prove the desired complexity result, we then successively reduce this variant to the standard Secure Set problem. At the same time, we show $\mathrm {\Sigma ^P_2}$-completeness for the exact variants of these problems, where we are interested in secure sets *exactly* of a certain size.

Membership in the class $\mathrm {\Sigma ^P_2}$ is rather obvious; in fact, [1] presents a polynomial-time reduction to Answer Set Programming [10] and thus shows this result implicitly. Together with our corresponding hardness result, it follows that Secure Set is $\mathrm {\Sigma ^P_2}$-complete, and it turns out that all the problem variants we consider in this paper are $\mathrm {\Sigma ^P_2}$-complete.

We thus complete the picture of the precise complexity of the Secure Set problem, and we also provide completeness results for variants of the problem that have already been proposed [22] but for which no complexity analysis has been performed so far. Our results underline that Secure Set is among the few rather natural problems in graph theory that are complete for the second layer of the polynomial hierarchy (like, e.g., Clique Coloring [25] or 2-Coloring Extension [28]). Moreover, $\mathrm {\Sigma ^P_2}$-hardness of Secure Set indicates that an efficient reduction to the Sat problem is not possible (unless the polynomial hierarchy collapses).

The second main contribution of our paper is a parameterized complexity analysis of Secure Set with treewidth as the parameter. We show that this problem is hard for the class $\mathrm {W[1]}$, which rules out a fixed-parameter tractable algorithm under commonly held complexity-theoretic assumptions. This result is rather surprising for two reasons: First, the problem is tractable on trees [22] and often problems that become easy on trees turn out to be fixed-parameter tractable when parameterized by treewidth.^1^ Second, this makes Secure Set one of the very few “subset problems” that are fixed-parameter tractable w.r.t. solution size but not w.r.t. treewidth. Problems with this kind of behavior are rather rare, as observed by Dom et al. [15].

Beside this parameterized hardness result, we also give an upper bound by showing that Secure Set is in the class $\mathrm {XP}$, which means that it can be solved in polynomial time on instances of bounded treewidth. We do so by providing an algorithm where the degree of the polynomial depends on the treewidth.

Finally, we present a positive result for the $\mathrm {\text {co-}NP}$-complete problem of checking whether a given set of vertices is secure in a graph: We provide an algorithm that solves the problem in linear time for graphs of bounded treewidth.

This paper is organized as follows: We first provide the necessary background in Sect. 2. Then we analyze the complexity of Secure Set in Sect. 3, where we show that this problem, along with several variants, is $\mathrm {\Sigma ^P_2}$-complete. In Sect. 4, we consider the parameterized complexity of Secure Set where treewidth is our parameter of interest. Section 5 concludes the paper with a discussion.

The present work extends a conference paper [4], which did not contain any results about the parameterized complexity of the considered problems. Beside the new results in Sect. 4, we also slightly modified some of the reductions that prove $\mathrm {\Sigma ^P_2}$-hardness so that they preserve bounded treewidth, which allows us to reuse them for our parameterized hardness proofs. We also added a reduction (which eliminates necessary vertices), which made one of the reductions (from the exact variant of the problem to the non-exact variant) from the previous paper redundant.

## Background

All graphs are undirected and simple unless stated otherwise. We denote the set of vertices and edges of a graph *G* by *V*(*G*) and *E*(*G*), respectively. We denote an undirected edge between vertices *u* and *v* as (*u*, *v*) or equivalently (*v*, *u*). It will be clear from the context whether an edge (*u*, *v*) is directed or undirected. Given a graph *G*, the *open neighborhood* of a vertex $v \in V(G)$, denoted by $N_G(v)$, is the set of all vertices adjacent to *v*, and $N_G[v] = N_G(v) \cup \{v\}$ is called the *closed neighborhood* of *v*. Let $S \subseteq V(G)$. We abuse notation by writing $N_G(S)$ and $N_G[S]$ to denote $\bigcup _{v \in S} N_G(v)$ and $\bigcup _{v \in S} N_G[v]$, respectively. If it is clear from the context which graph is meant, we write $N(\cdot )$ and $N[\cdot ]$ instead of $N_G(\cdot )$ and $N_G[\cdot ]$, respectively.

### Definition 1

Given a graph *G*, a set $S \subseteq V(G)$ is *secure in G* if for each $X \subseteq S$ it holds that ${|N[X] \cap S |} \ge {|N[X] {\setminus } S |}$.

**Fig. 1:**
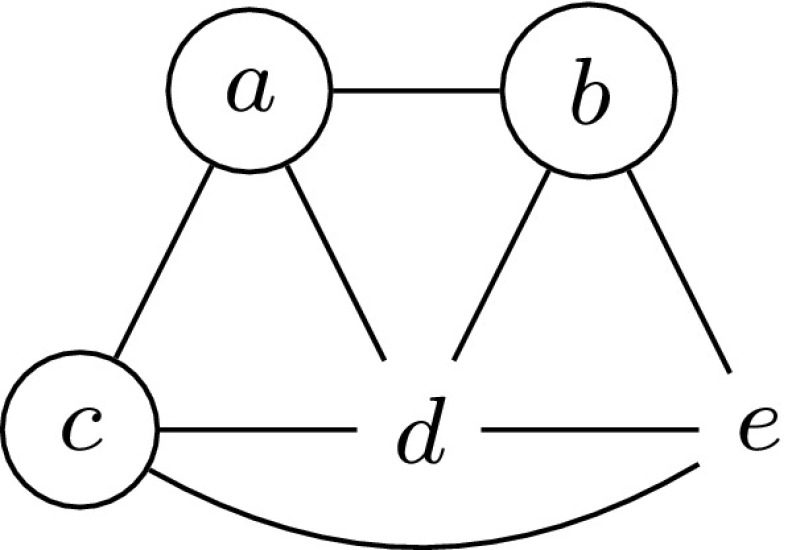
A graph with a minimum non-empty secure set indicated by *circled vertices*

We often write “*S* is secure” instead of “*S* is secure in *G*” if it is clear from the context which graph is meant. By definition, the empty set is secure in any graph. Thus, in the following decision problems we ask for secure sets of size at least 1. The following is our main problem: 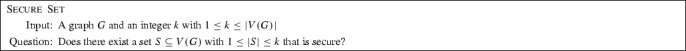 Figure 1 shows a graph together with a minimum non-empty secure set $S = \{a,b,c\}$. Observe that for any $X \subseteq S$ the condition ${|N[X] \cap S |} \ge {|N[X] {\setminus } S |}$ is satisfied.

Note that the well-known Defensive Alliance problem is a special case of Secure Set where only those subsets *X* of *S* are considered that have size 1. For example, in Fig. 1, the set $S' = \{a,b\}$ is a defensive alliance as ${|N[v] \cap S' |} \ge {|N[v] {\setminus } S' |}$ holds for each $v \in S'$, but $S'$ is not a secure set, since for $X' = S'$ it holds that ${|N[X'] \cap S' |} < {|N[X'] {\setminus } S' |}$.

We now define three variants of the Secure Set problem that we require in our proofs. ${\textsc {Secure}\,\textsc {Set}^\textsc {F}}$ generalizes the Secure Set problem by designating some “forbidden” vertices that may never be in any solution. This variant can be formalized as follows: 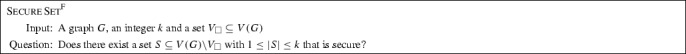 ${\textsc {Secure}\,\textsc {Set}^\textsc {FN}}$ is a further generalization that, in addition, allows “necessary” vertices to be specified that must occur in every solution. 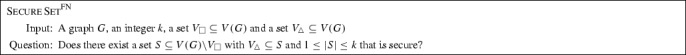 Finally, we introduce the generalization ${\textsc {Secure}\,\textsc {Set}^\textsc {FNC}}$. Here we may state pairs of “complementary” vertices where each solution must contain exactly one element of every such pair. 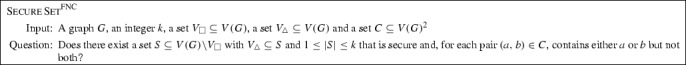 For our results on structural parameters, we need a way to represent the structure of a ${\textsc {Secure}\,\textsc {Set}^\textsc {FNC}}$ instance by a graph that augments *G* with the information in *C*:

### Definition 2

Let *I* be a ${\textsc {Secure}\,\textsc {Set}^\textsc {FNC}}$ instance, let *G* be the graph in *I* and let *C* the set of complementary vertex pairs in *I*. By the *primal graph* of *I* we mean the graph $G'$ with $V(G') = V(G)$ and $E(G') = E(G) \cup C$.

While the Secure Set problem asks for secure sets of size *at most* *k*, we also consider the Exact Secure Set problem that concerns secure sets of size *exactly* *k*. Note that a secure set may become insecure by adding or removing elements, so this is a non-trivial problem variant. Analogously, we also define exact versions of the three generalizations of Secure Set presented above.

When the task is not to find secure sets but to verify whether a given set is secure, the following problem is of interest: 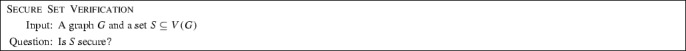 This problem is known to be $\mathrm {\text {co-}NP}$-complete [21].

**Fig. 2:**
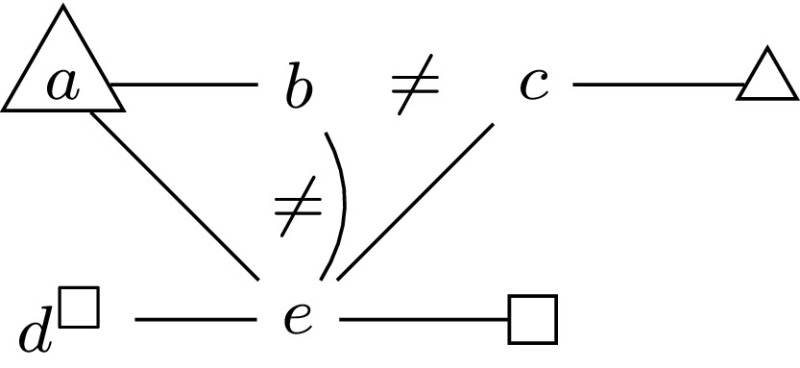
Illustration of forbidden, necessary and complementary vertices

In this paper’s figures, we often indicate necessary vertices by means of a triangular node shape, and forbidden vertices by means of either a square node shape or a superscript square in the node name. If two vertices are complementary, we often express this in the figures by putting a $\ne$ sign between them. For example, in Fig. 2, the vertices *b* and *c* are complementary and occur in no solution together; also the vertices *b* and *e* are complementary. Note, however, that by putting a $\ne$ sign between two vertices we do not mean to express that there is an edge between them. For instance, there is no edge between *b* and *c*, but there is an edge between *b* and *e*, which is explicitly drawn. The vertex *a* and the “anonymous” vertex adjacent to *c* are necessary and occur in every solution; $d^\square$ and the “anonymous” vertex adjacent to *e* are forbidden and occur in no solution. In this figure, the unique minimum non-empty secure set satisfying the conditions of forbidden, necessary and complementary vertices consists of *a*, *b* and the “anonymous” necessary vertex adjacent to *c*.

The following terminology will be helpful: We often use the terms *attackers* and *defenders* of a subset *X* of a secure set candidate *S*. By these we mean the sets $N[X] {\setminus } S$ and $N[X] \cap S$, respectively. To show that a subset *X* of a secure set candidate *S* is *not* a witness to *S* being insecure, we sometimes employ the notion of a *defense* of *X* w.r.t. *S*, which assigns to each attacker a dedicated defender: If we are able to find an injective mapping $\mu : N[X] {\setminus } S \rightarrow N[X] \cap S$, then obviously ${|N[X] {\setminus } S |} \le {|N[X] \cap S |}$, and we call $\mu$ a *defense* of *X* w.r.t. *S*. Given such a defense $\mu$, we say that a defender *d* *repels* an attack on *X* by an attacker *a* whenever $\mu (a) = d$. Consequentially, when we say that a set of defenders *D* *can repel* attacks on *X* from a set of attackers *A*, we mean that there is a defense that assigns to each element of *A* a dedicated defender in *D*.

To warm up, we make some easy observations that we will use in our proofs. First, for every set *R* consisting of a majority of neighbors of a vertex *v*, whenever *v* is in a secure set, also some element of *R* must be in it:

### Observation 1

Let *S* be a secure set in a graph, let $v \in S$ and let $R \subseteq N(v)$. If ${|R |} > \frac{1}{2} N[v]$, then *S* contains an element of *R*.

### Proof

Suppose that ${|R |} > \frac{1}{2} {|N[v] |}$ and *S* contains no element of *R*. Since all elements of *R* attack *v*, ${|N[v] {\setminus } S |} > \frac{1}{2} {|N[v] |}$. Hence $2 {|N[v] {\setminus } S |} > {|N[v] |} = {|N[v] \cap S |} + {|N[v] {\setminus } S |}$, and we obtain the contradiction ${|N[v] {\setminus } S |} > {|N[v] \cap S |}$. $\square$

Next, if one half of the neighbors of an element *v* of a secure set attacks *v*, then the other half of the neighbors must be in the secure set:

### Observation 2

Let *S* be a secure set in a graph, let $v \in S$ and let *N*(*v*) be partitioned into two equal-sized sets *A*, *D*. If $A \cap S = \emptyset$, then $D \subseteq S$.

### Proof

Since *N*(*v*) is partitioned into *A* and *D* such that $A \cap S = \emptyset$, we get $N(v) \cap S = D \cap S$. If some element of *D* is not in *S*, then $D \cap S \subset D$ and $A \subset N[v] {\setminus } S$. By ${|D |} = {|A |}$, we get ${|D \cap S |} + 2 \le {|N[v] {\setminus } S |}$. From ${|N[v] \cap S |} = 1 + {|N(v) \cap S |} = 1 + {|D \cap S |}$ we now obtain the contradiction ${|N[v] \cap S |} < {|N[v] {\setminus } S |}$. $\square$

In particular, if half of the neighbors of *v* are forbidden, then *v* can only be in a given secure set if all non-forbidden neighbors are also in the secure set.

Finally, we recapitulate some background from complexity theory. The class $\mathrm {\Sigma ^P_2}$ is the class of problems that are solvable in polynomial time by a nondeterministic Turing machine that has access to an $\mathrm {NP}$ oracle. The canonical problem complete for this class is $\textsc {Qsat}_\textsc {2}$, which asks, given a formula $\exists x_1 \dots \exists x_{n_x} \forall y_1 \dots \forall y_{n_y} \psi$, where $\psi$ is a propositional 3-DNF formula, whether there is a truth assignment to the $x_i$ variables such that for all truth assignments to the $y_i$ variables $\psi$ evaluates to true.

In parameterized complexity theory [13, 16, 19, 26], we study problems that consist not only of an input and a question, but also of some parameter of the input that is represented as an integer. A problem is in the class $\mathrm {FPT}$ (“fixed-parameter tractable”) if it can be solved in time $f(k) \cdot n^c$, where *n* is the input size, *k* is the parameter, *f* is a computable function that only depends on *k*, and *c* is a constant that does not depend on *k* or *n*. We call such an algorithm an *FPT algorithm*, and we call it *fixed-parameter linear* if $c=1$. Similarly, a problem is in the class $\mathrm {XP}$ (“slice-wise polynomial”) if it can be solved in time $f(k) \cdot n^{g(k)}$, where *f* and *g* are computable functions. Note that here the degree of the polynomial may depend on *k*, so such algorithms are generally slower than FPT algorithms. For the class $\mathrm {W[1]}$ it holds that $\mathrm {FPT}\subseteq \mathrm {W[1]}\subseteq \mathrm {XP}$, and it is commonly believed that the inclusions are proper, i.e., $\mathrm {W[1]}$-hard problems do not admit FPT algorithms. $\mathrm {W[1]}$-hardness of a problem can be shown using parameterized reductions, which are reductions that run in FPT time and produce an equivalent instance whose parameter is bounded by a function of the original parameter.

For problems whose input can be represented as a graph, one important parameter is *treewidth*, which is a structural parameter that, roughly speaking, measures the “tree-likeness” of a graph. It is defined by means of tree decompositions, originally introduced in [27]. The intuition behind tree decompositions is to obtain a tree from a (potentially cyclic) graph by subsuming multiple vertices under one node and thereby isolating the parts responsible for cyclicity.

### Definition 3

A *tree decomposition* of a graph *G* is a pair $\mathcal {T}= (T,\chi )$ where *T* is a (rooted) tree and $\chi : V(T) \rightarrow 2^{V(G)}$ assigns to each node of *T* a set of vertices of *G* (called the node’s *bag*), such that the following conditions are met:

1. For every vertex $v \in V(G)$, there is a node $t \in V(T)$ such that $v \in \chi (t)$.
2. For every edge $(u,v) \in E(G)$, there is a node $t \in V(T)$ such that $\{u,v\} \subseteq \chi (t)$.
3. For every $v \in V(G)$, the subtree of *T* induced by $\{t \in V(T) \mid v \in \chi (t)\}$ is connected.

We call $\max _{t \in V(T)} |\chi (t) |- 1$ the *width* of $\mathcal {T}$. The *treewidth* of a graph is the minimum width over all its tree decompositions.

In general, constructing an optimal tree decomposition (i.e., a tree decomposition with minimum width) is intractable [2]. However, the problem is solvable in linear time on graphs of bounded treewidth (specifically in time $w^{\mathcal {O}(w^3)} \cdot n$, where *w* is the treewidth) [6] and there are also heuristics that offer good performance in practice [8, 14].

In this paper we will consider so-called *nice* tree decompositions:

### Definition 4

A tree decomposition $\mathcal {T} = (T,\chi )$ is *nice* if each node $t \in V(T)$ is of one of the following types:

1. Leaf node: The node *t* has no child nodes.
2. Introduce node: The node *t* has exactly one child node $t'$ such that $\chi (t) {\setminus } \chi (t')$ consists of exactly one element.
3. Forget node: The node *t* has exactly one child node $t'$ such that $\chi (t') {\setminus } \chi (t)$ consists of exactly one element.
4. Join node: The node *t* has exactly two child nodes $t_1$ and $t_2$ with $\chi (t) = \chi (t_1) = \chi (t_2)$.

Additionally, the bags of the root and the leaves of *T* are empty.

A tree decomposition of width *w* for a graph with *n* vertices can be transformed into a nice one of width *w* with $\mathcal {O}(wn)$ nodes in fixed-parameter linear time [23].

**Fig. 3:**
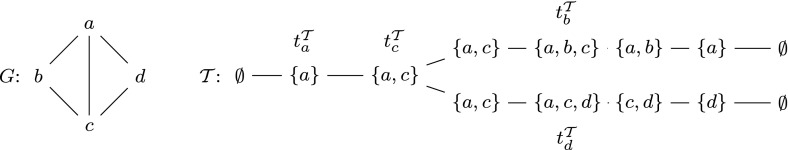
A graph *G* and a nice tree decomposition $\mathcal {T}$ of *G* rooted at the leftmost node

For any tree decomposition $\mathcal {T}$ and an element *v* of some bag in $\mathcal {T}$, we use the notation $t_v^\mathcal {T}$ to denote the unique “topmost node” whose bag contains *v* (i.e., $t_v^\mathcal {T}$ does not have a parent whose bag contains *v*). Figure 3 depicts a graph and a nice tree decomposition, where we also illustrate the $t^\mathcal {T}_v$ notation.

When we speak of the treewidth of an instance of Secure Set, ${\textsc {Secure}\,\textsc {Set}^\textsc {F}}$, ${\textsc {Secure}\,\textsc {Set}^\textsc {FN}}$, Exact Secure Set, ${\textsc {Exact}\,\textsc {Secure}\,\textsc {Set}^\textsc {F}}$ or ${\textsc {Exact}\,\textsc {Secure}\,\textsc {Set}^\textsc {FN}}$, we mean the treewidth of the graph in the instance. For an instance of ${\textsc {Secure}\,\textsc {Set}^\textsc {FNC}}$ or ${\textsc {Exact}\,\textsc {Secure}\,\textsc {Set}^\textsc {FNC}}$, we mean the treewidth of the primal graph.

## Complexity of the Secure Set Problem

This section is devoted to proving the following theorem:

### Theorem 1

The following problems are all $\mathrm {\Sigma ^P_2}$-complete: Secure Set, Exact Secure Set, ${\textsc {Secure}\,\textsc {Set}^\textsc {F}}$, ${\textsc {Exact}\,\textsc {Secure}\,\textsc {Set}^\textsc {F}}$, ${\textsc {Secure}\,\textsc {Set}^\textsc {FN}}$, $\textsc {Exact}\,\textsc {Secure}\,\textsc {Set}^\textsc {FN}$, ${\textsc {Secure}\,\textsc {Set}^\textsc {FNC}}$ and ${\textsc {Exact}\,\textsc {Secure}\,\textsc {Set}^\textsc {FNC}}$.

We prove this by providing a chain of polynomial reductions from $\textsc {Qsat}_\textsc {2}$ to the problems under consideration.

### Hardness of Secure Set with Forbidden, Necessary and Complementary Vertices

#### Lemma 1

${\textsc {Secure}\,\textsc {Set}^\textsc {FNC}}$ and ${\textsc {Exact}\,\textsc {Secure}\,\textsc {Set}^\textsc {FNC}}$ are $\mathrm {\Sigma ^P_2}$-hard.

#### Proof

We reduce from $\textsc {Qsat}_\textsc {2}$ to ${\textsc {Secure}\,\textsc {Set}^\textsc {FNC}}$. This also proves $\mathrm {\Sigma ^P_2}$-hardness for the exact variant because our reduction makes sure that all solutions of the ${\textsc {Secure}\,\textsc {Set}^\textsc {FNC}}$ instance have the same size. We are given a quantified Boolean formula $\varphi = \exists x_1 \dots \exists x_{n_x} \forall y_1 \dots \forall y_{n_y} \psi$, where $\psi$ is in 3-DNF and contains $n_t$ terms. We assume that no term contains both a variable and its complement (since such a term can never be satisfied) and that each term contains at least one universally quantified variable (since $\varphi$ is trivially true otherwise).

**Fig. 4:**
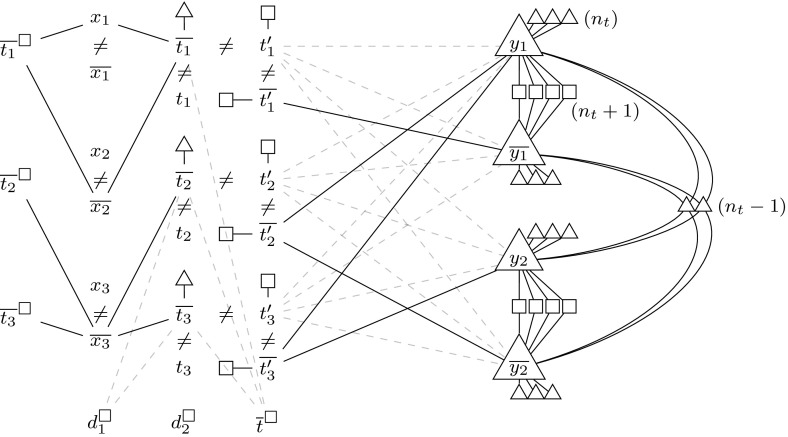
Graph corresponding to the $\textsc {Qsat}_\textsc {2}$ formula $\exists x_1 \exists x_2 \exists x_3\; \forall y_1 \forall y_2\; \big ((\lnot x_1 \wedge x_2 \wedge y_1) \vee (x_3 \wedge \lnot y_1 \wedge y_2) \vee (x_3 \wedge \lnot y_1 \wedge \lnot y_2)\big )$. To avoid clutter, we omit labels for the vertices from $Y_\triangle$, $Y'_\triangle$, $Y_\square$, $\overline{T}_\triangle$, $T'_\square$ and $\overline{T'}_\square$, and we draw some edges in a *dashed style*

We construct an instance $(G,k,V_\triangle ,V_\square ,C)$ of ${\textsc {Secure}\,\textsc {Set}^\textsc {FNC}}$ in the following. For an illustration, see Fig. 4. We define a graph *G* by choosing the union of the following sets as *V*(*G*):

$$\begin{aligned} X ={}&\{x_1, \dots , x_{n_x} \}&\overline{X}={}&\{\overline{x_1}, \dots , \overline{x_{n_x}} \} \\ Y ={}&\{y_1, \dots , y_{n_y} \}&\overline{Y}={}&\{\overline{y_1}, \dots , \overline{y_{n_y}} \} \\ Y_\triangle ={}&\left\{ y_{i,j}^\triangle , \overline{y_{i,j}}^\triangle \mid 1 \le i \le n_y,\; 1 \le j \le n_t \right\}&Y'_\triangle ={}&\left\{ y_j^\triangle \mid 1 \le j \le n_t - 1 \right\} \\ Y_\square ={}&\left\{ y_{i,j}^\square \mid 1 \le i \le n_y,\; 1 \le j \le n_t+1 \right\}&H ={}&\left\{ d_1^\square , d_2^\square , \overline{t}^\square \right\} \\ T ={}&\{t_1, \dots , t_{n_t}\}&\overline{T}={}&\left\{ \overline{t_1}, \dots , \overline{t_{n_t}} \right\} \\ \overline{T}_\square ={}&\left\{ \overline{t_1}^\square , \dots , \overline{t_{n_t}}^\square \right\}&\overline{T}_\triangle ={}&\left\{ \overline{t_1}^\triangle , \dots , \overline{t_{n_t}}^\triangle \right\} \\ T'={}&\{t_1', \dots , t_{n_t}'\}&\overline{T'}={}&\{\overline{t'_1}, \dots , \overline{t'_{n_t}} \} \\ T'_\square ={}&\left\{ t_1'^\square , \dots , t_{n_t}'^\square \right\}&\overline{T'}_\square ={}&\left\{ \overline{t_1'}^\square , \dots , \overline{t_{n_t}'}^\square \right\} \end{aligned}$$

Next we define the set of edges. In the following, whenever we sloppily speak of a literal in the context of the graph *G*, we mean the vertex corresponding to that literal (i.e., some $x_i$, $\overline{x_i}$, $y_i$ or $\overline{y_i}$), and we proceed similarly for terms. Furthermore, when we are dealing with a (vertex corresponding to a) literal *l*, then $\overline{l}$ shall denote the (vertex corresponding to the) complement of *l*. For any term $t_i$, let $L_X(t_i)$ and $L_Y(t_i)$ denote the set of existentially and universally quantified literals, respectively, in $t_i$.

$$\begin{aligned} E(G) ={}&\left\{ \left( \overline{t_i},\overline{t}^\square \right) , \left( \overline{t_i},\overline{t_i}^\triangle \right) , \left( t_i',t_i'^\square \right) , \left( \overline{t_i'},\overline{t_i'}^\square \right) \mid t_i \in T\right\} \cup \left( T'\times (Y \cup \overline{Y})\right) \\&{}\cup {} \left\{ \left( \overline{l},\overline{t_i}^\square \right) , (\overline{l},\overline{t_i}) \mid t_i \in T,\; l \in L_X(t_i)\right\} \\&{}\cup {} \left\{ \left( \overline{l},\overline{t_i'}\right) \mid t_i \in T,\; l \in L_Y(t_i)\right\} \\&{}\cup {} \left\{ \left( d_1^\square ,\overline{t_i}\right) \mid t_i \in T,\; {|L_X(t_i) |} \le 1\right\} \cup \left\{ \left( d_2^\square ,\overline{t_i}\right) \mid t_i \in T,\; L_X(t_i) = \emptyset \right\} \\&{}\cup {} \left\{ \left( y_i,y_{i,j}^\triangle \right) , \left( \overline{y_i},\overline{y_{i,j}}^\triangle \right) \mid 1 \le i \le n_y,\; 1 \le j \le n_t\right\} \\&{}\cup {} \left\{ \left( y_i,y_{i,j}^\square \right) , \left( \overline{y_i},y_{i,j}^\square \right) \mid y_{i,j}^\square \in Y_\square \right\} \cup \left( Y'_\triangle \times (Y \cup \overline{Y})\right) \end{aligned}$$

Finally, we define

$$\begin{aligned} V_\triangle ={}&Y \cup \overline{Y}\cup Y_\triangle \cup Y'_\triangle \cup \overline{T}_\triangle , \quad V_\square = Y_\square \cup \overline{T}_\square \cup T'_\square \cup \overline{T'}_\square \cup H,\\ C ={}&\{(x_i,\overline{x_i}) \mid 1 \le i \le n_x\} \cup \left\{ \left( t_i,\overline{t_i}\right) , \left( \overline{t_i},t_i'\right) , \big (t_i', \overline{t_i'}\big ) \mid 1 \le i \le n_t\right\} , \end{aligned}$$

and $$k = {|V_\triangle |} + n_x + 2n_t$$.

The following observations are crucial: Elements of $X \cup \overline{X}$ are only adjacent to vertices from $\overline{T}_\square$ (which are forbidden) and $\overline{T}$. For any *i*, each element of $X \cup \overline{X}$ is adjacent to $\overline{t_i}^\square \in \overline{T}_\square$ iff it is adjacent to $\overline{t_i} \in \overline{T}$. Furthermore, for any *i*, *j*, if $x_i$ or $\overline{x_i}$ is adjacent to $\overline{t_j}$, then setting the variable $x_i$ to $\text {true}$ or $\text {false}$, respectively, falsifies the term $t_j$. Finally, for any *i*, *j*, if $y_i$ or $\overline{y_i}$ is adjacent to $\overline{t_j'}$, then setting the variable $y_i$ to $\text {true}$ or $\text {false}$, respectively, falsifies the term $t_j$.

The intuition is that the complementary pairs $(x_i,\overline{x_i})$ guess a truth assignment to the existentially quantified variables. We now need to check if such a truth assignment has the property that the formula $\psi$ is true for all extensions of this assignment to the universally quantified variables. Trying out all these extensions amounts to going through all subsets of a solution candidate and comparing the numbers of attackers and defenders.

To illustrate, let *S* be a solution candidate (i.e., a set of vertices) and suppose *S* satisfies the conditions on forbidden, necessary and complementary vertices. We denote the truth assignment to $x_1,\dots ,x_{n_x}$ encoded in *S* by $I_S$. Moreover, let *R* be a subset of *S* containing either $y_j$ or $\overline{y_j}$ for each universally quantified variable $y_j$. We denote the extension of $I_S$ to $y_1,\dots ,y_{n_y}$ encoded in *R* by $I_{S,R}$. For any term $t_i$ that is falsified already by $I_S$, the vertex $t_i'$ attacks all vertices $y_j$ and $\overline{y_j}$. At the same time, for any term $t_i$ that is not falsified by $I_S$, the vertex $\overline{t_i'}$ attacks $y_j$ or $\overline{y_j}$ if setting the variable $y_j$ to $\text {true}$ or $\text {false}$, respectively, falsifies $t_i$. Hence, the number of attacks from vertices of the form $t_i'$ or $\overline{t_i'}$ on *R* is exactly the number of terms that are falsified by $I_{S,R}$. With the help of the vertices in $Y'_\triangle$, we can afford up to $n_t-1$ falsified terms, but if we falsify all $n_t$ terms, then *R* is a witness that *S* is not secure.

The ${\textsc {Secure}\,\textsc {Set}^\textsc {FNC}}$ instance $(G,k,V_\triangle ,V_\square ,C)$ can be constructed in time polynomial in the size of $\varphi$. We claim that $\varphi$ is true iff $(G,k,V_\triangle ,V_\square ,C)$ is a positive instance of ${\textsc {Secure}\,\textsc {Set}^\textsc {FNC}}$.

“*Only if*” *direction* If $\varphi$ is true, then there is an assignment *I* to $x_1, \dots , x_{n_x}$ such that, for all assignments extending *I* to $y_1, \dots , y_{n_y}$, some term in $\psi$ is satisfied. We define a set

$$\begin{aligned} S ={}&V_\triangle \cup \{x_i \in X \mid I(x_i) = \text {true}\} \cup \{\overline{x_i} \in \overline{X}\mid I(x_i) = \text {false}\} \\&{}\cup {} \left\{ \overline{t_i} \in \overline{T},\; \overline{t_i'} \in \overline{T'}\mid \text {there is some } l \in L_X(t_i) \text { such that } I \not \models l\right\} \\&{}\cup {} \left\{ t_i \in T,\; t_i' \in T'\mid \text {for all } l \in L_X(t_i) \text { it holds that } I \models l\right\} . \end{aligned}$$

We observe that ${|S |} = k$, $V_\square \cap S = \emptyset$, $V_\triangle \subseteq S$, and that for any $(a,b) \in C$ it holds that $a \in S$ iff $b \notin S$. By construction, whenever some element of $X \cup \overline{X}$ is in *S*, then all its neighbors in $\overline{T}$ are in *S*; and whenever some $\overline{t_i}$ is in *S*, then some neighbor of $\overline{t_i}$ in $X \cup \overline{X}$ is in *S*.

We claim that *S* is a secure set in *G*. Let *R* be an arbitrary subset of *S*. We show that *R* has at least as many defenders as attackers by constructing a defense, which assigns to each attacker of *R* a dedicated defender in $N[R] \cap S$. We distinguish cases regarding the origins of the attacks on *R*.

- We repel each attacker $\overline{t_i}^\square \in \overline{T}_\square$ using $\overline{t_i}$. Since $\overline{t_i}^\square$ attacks *R*, *R* must contain some element of $X \cup \overline{X}$ that is adjacent to $\overline{t_i}^\square$ and thus also to $\overline{t_i}$, so $\overline{t_i} \in N[R] \cap S$.
- Each attacker from $X \cup \overline{X}\cup \{d_1^\square ,d_2^\square \}$ is adjacent to some $\overline{t_i} \in \overline{T}\cap R$. We repel that attacker using $\overline{t_i}^\triangle$, which is adjacent to $\overline{t_i}$. Note that it cannot be the case that $\overline{t_i}$ is attacked by more than one vertex in $X \cup \overline{X}\cup \{d_1^\square ,d_2^\square \}$ because $\overline{t_i}$ has exactly two neighbors from that set and would not be in *S* if neither of these neighbors was in *S*.
- If $\overline{t}^\square$ attacks *R*, then it attacks at least one element of $\overline{T}\cap R$, which is adjacent to some element of $X \cup \overline{X}$ that is also in *S*. We repel $\overline{t}^\square$ using any such element of $X \cup \overline{X}$.
- Any attack from some $\overline{t_i} \in \overline{T}$ on *R* must be on $\overline{t_i}^\triangle$. Since $\overline{t_i} \notin S$, $\overline{t_i}^\triangle$ is not consumed for repelling an attack on $\overline{t_i}$, so we repel $\overline{t_i}$ with $\overline{t_i}^\triangle$.
- If some $t_i'^\square \in T'_\square$ attacks *R* (by attacking $t_i'$), we repel $t_i'^\square$ with $t_i'$.
- Analogously, we repel each attacker $\overline{t_i'}^\square \in \overline{T'}_\square$ with $\overline{t_i'}$.
- If, for some *i* with $1 \le i \le n_y$, the vertices $y_{i,j}^\square$ for $1 \le j \le n_t+1$ attack *R*, then we distinguish the following cases: If $y_i$ is in *R*, then the adjacent vertices $y_{i,j}^\triangle$ for $1 \le j \le n_t$ are in the neighborhood of *R*, too. We then repel each $y_{i,j}^\square$ with $y_{i,j}^\triangle$ for $1 \le j \le n_t$, and we repel $y_{i,n_t+1}^\square$ with $y_i$. Otherwise, $\overline{y_i}$ is in *R*, and we proceed symmetrically using $\overline{y_{i,j}}^\triangle$ and $\overline{y_i}$ as dedicated defenders.
- In order to account for attacks from $T'\cup \overline{T'}$ on *R*, we distinguish two cases.
  - If, for some *i* with $1 \le i \le n_y$, both $y_i$ and $\overline{y_i}$ are in *R*, then, in the step before, we have repelled each $y_{i,j}^\square$ with the respective $y_{i,j}^\triangle$ or $y_i$, but all $\overline{y_{i,j}}^\triangle$ are still free. These vertices can repel all attacks from $T'\cup \overline{T'}$, as there are at most $n_t$ such attacks.
  - Otherwise we show that there are at most $n_t - 1$ attacks from $T'\cup \overline{T'}$, and they can be repelled using $Y'_\triangle$. Consider the (partial) assignment *J* that assigns the same values to the variables $x_1, \dots , x_{n_x}$ as the assignment *I* above, and, for any variable $y_i$, sets $y_i$ to $\text {true}$ or $\text {false}$ if *R* contains the vertex $y_i$ or $\overline{y_i}$, respectively. By assumption we know that our assignment to $x_1, \dots , x_{n_x}$ is such that for all assignments to $y_1, \dots , y_{n_y}$ some term $t_i$ in $\psi$ is true. In particular, it must therefore hold that *J* falsifies no existentially quantified literal in $t_i$. Then, by construction of *S*, the vertex $\overline{t_i'}$ is not in *S*. We also know that *J* falsifies no universally quantified literal in $t_i$. But then the vertices from $Y \cup \overline{Y}$ adjacent to the vertex $\overline{t_i'}$ are not in *R* due to our construction of *J*, so $\overline{t_i'}$ does not attack any vertex in *R*. From this it follows that there are at most $n_t - 1$ attacks from $T'\cup \overline{T'}$ on *R*. We can repel all these attacks using the vertices $y_1^\triangle , \dots , y_{n_t-1}^\triangle$.

This allows us to conclude ${|N[R] \cap S |} \ge {|N[R] {\setminus } S |}$. Therefore *S* is secure.

“*If*” *direction* Suppose *S* is a secure set in *G* satisfying the conditions regarding forbidden, necessary and complementary vertices. First observe that ${|S |} = k$ because the complementary vertex pairs make sure that *S* contains exactly half of $V(G) {\setminus } (V_\triangle \cup V_\square )$.

If *S* contains some $l \in X \cup \overline{X}$, then $N(l) \cap \overline{T}\subseteq S$ by Observation 2. If *S* contains some $\overline{t_i} \in \overline{T}$, then $\overline{t_i}$ must be adjacent to some element of $X \cup \overline{X}$ that is also in *S* by Observation 1.

We construct an interpretation *I* on the variables $x_1, \dots , x_{n_x}$ that sets exactly those $x_i$ to true where the corresponding vertex $x_i$ is in *S*, and we claim that for each extension of *I* to the universally quantified variables there is a satisfied term in $\psi$. To see this, suppose to the contrary that some assignment *J* to all variables extends *I* but falsifies all terms in $\psi$. Then we define a set *R* consisting of all vertices $y_i$ such that $J(y_i) = \text {true}$, all vertices $\overline{y_i}$ such that $J(y_i) = \text {false}$, and all vertices in $(T'\cup \overline{T'}) \cap S$ that are adjacent to these vertices $y_i$ or $\overline{y_i}$. We show that this contradicts *S* being secure: Clearly, *R* is a subset of *S* and has ${|R |}$ defenders due to itself, $n_t - 1$ defenders due to $Y'_\triangle$, and $n_y \cdot n_t$ defenders due to $N(R) \cap Y_\triangle$. This amounts to ${|N[R] \cap S |} = {|R |} + n_t - 1 + n_y \cdot n_t$. On the other hand, there are $n_t$ attacks on *R* from $T'\cup \overline{T'}$. This is because for any term $t_i$ in $\psi$ one of the following cases applies:

- The term $t_i$ is falsified already by *I*. Then $\overline{t_i'} \in S$ and thus $t_i' \notin S$. The vertex $t_i'$, however, is adjacent to every element of $Y \cup \overline{Y}$, so it attacks *R*.
- The term $t_i$ is not falsified by *I* but by *J*. Then $\overline{t_i'} \notin S$, and $L_Y(t_i)$ contains some literal *l* with $\overline{l} \in N(\overline{t_i'})$ and $J \models \overline{l}$, so $\overline{l}$ is in *R* and attacked by $\overline{t_i'}$.

In addition to these $n_t$ attackers, *R* has ${|R \cap (T'\cup \overline{T'}) |}$ attackers in $N(R) \cap (T'_\square \cup \overline{T'}_\square )$, as well as $n_y \cdot (n_t+1)$ attackers in $Y_\square$. As ${|R |} = n_y + {|R \cap (T'\cup \overline{T'}) |}$, we obtain in total

$$\begin{aligned} {|N[R] {\setminus } S |}= & {} n_t + {|R \cap (T'\cup \overline{T'}) |} + n_y \cdot (n_t+1)\\= & {} {|R |} + n_t + n_y \cdot n_t > {|N[R] \cap S |}. \end{aligned}$$

This contradicts *S* being secure, so for each extension of *I* to the universally quantified vertices, $\psi$ is true; hence $\varphi$ is true. $\square$

### Hardness of Secure Set with Forbidden and Necessary Vertices

Next we present a transformation $\tau ^\text {FNC}$ that eliminates complementary vertex pairs by turning a ${\textsc {Secure}\,\textsc {Set}^\textsc {FNC}}$ instance into an equivalent ${\textsc {Secure}\,\textsc {Set}^\textsc {FN}}$ instance. Along with $\tau ^\text {FNC}$, we define a function $\sigma _{I}^\text {FNC}$, for each ${\textsc {Secure}\,\textsc {Set}^\textsc {FNC}}$ instance *I*, such that the solutions of *I* are in a one-to-one correspondence with those of $\tau ^\text {FNC}(I)$ in such a way that any two solutions of *I* have the same size iff the corresponding solutions of $\tau ^\text {FNC}(I)$ have the same size. We use these functions to obtain a polynomial-time reduction from ${\textsc {Secure}\,\textsc {Set}^\textsc {FNC}}$ to ${\textsc {Secure}\,\textsc {Set}^\textsc {FN}}$ as well as from ${\textsc {Exact}\,\textsc {Secure}\,\textsc {Set}^\textsc {FNC}}$ to ${\textsc {Exact}\,\textsc {Secure}\,\textsc {Set}^\textsc {FN}}$.

**Fig. 5:**
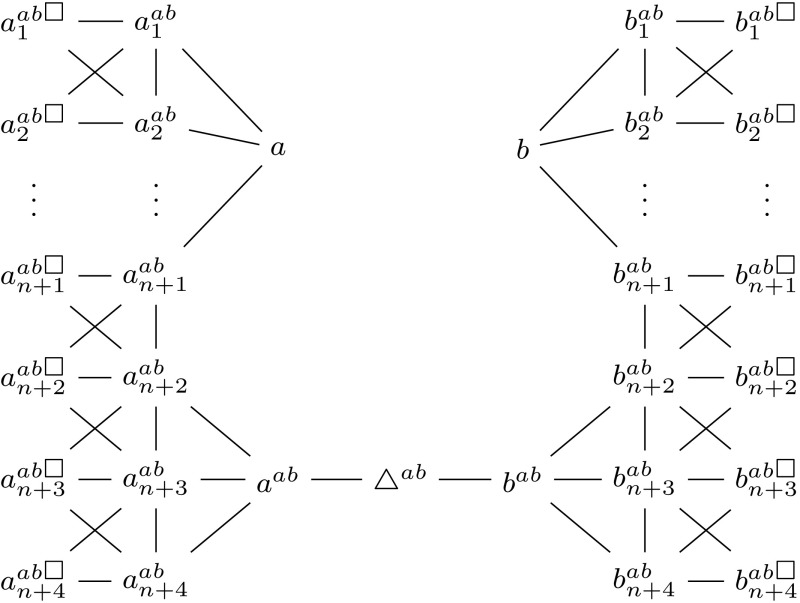
Gadget for a pair of complementary vertices (*a*, *b*) in the reduction from ${\textsc {Secure}\,\textsc {Set}^\textsc {FNC}}$ to ${\textsc {Secure}\,\textsc {Set}^\textsc {FN}}$. The vertices *a* and *b* may have additional neighbors from the original graph

Before we formally define our reduction, we briefly describe the underlying intuition. The gadget in Fig. 5 is added for every complementary pair (*a*, *b*). It is constructed in such a way that every solution must either contain all of $\{a,a^{ab},a^{ab}_1,\dots ,a^{ab}_{n+4}\}$ or none of them, and the same holds for $\{b,b^{ab},b^{ab}_1,\dots ,b^{ab}_{n+4}\}$. By making the vertex $\triangle ^{ab}$ necessary, every solution must contain one of these two sets. At the same time, the bound on the solution size makes sure that we cannot afford to take both sets for any complementary pair.

#### Definition 5

We define a function $\tau ^\text {FNC}$, which assigns a ${\textsc {Secure}\,\textsc {Set}^\textsc {FN}}$ instance to each ${\textsc {Secure}\,\textsc {Set}^\textsc {FNC}}$ instance $I = (G,k,V_\square ,V_\triangle ,C)$. For this, we use *n* to denote ${|V(G) |}$ and first define a function $\sigma _{I}^\text {FNC} : x \mapsto x + {|C |} \cdot (n+6)$. For each $(a,b) \in C$, we introduce new vertices $a^{ab}$, $b^{ab}$ and $\triangle ^{ab}$ as well as, for any $x \in \{a,b\}$, sets of new vertices $Y^{ab}_{x{\Circle }} = \{x^{ab}_1, \dots , x^{ab}_{n+1}\}$, $Z^{ab}_{x{\Circle }} = \{x^{ab}_{n+2}, x^{ab}_{n+3}, x^{ab}_{n+4}\}$, $Y^{ab}_{x\square } = \{x^{ab\square }_1, \dots , x^{ab\square }_{n+1}\}$ and $Z^{ab}_{x\square } = \{x^{ab\square }_{n+2}, x^{ab\square }_{n+3}, x^{ab\square }_{n+4}\}$. We use the notation $u \oplus v$ to denote the set of edges $\{(u,v),$ $(u,u^\square ),$ $(v,v^\square ),$ $(u,v^\square ),$ $(v,u^\square )\}$. Now we define the ${\textsc {Secure}\,\textsc {Set}^\textsc {FN}}$ instance $\tau ^\text {FNC}(I) = (G',k',V_\square ',V_\triangle ')$, where $k' = \sigma _{I}^\text {FNC}(k)$, $V_\square ' = V_\square \cup \bigcup _{(a,b) \in C} (Y^{ab}_{a\square } \cup Y^{ab}_{b\square } \cup Z^{ab}_{a\square } \cup Z^{ab}_{b\square })$, $V_\triangle ' = V_\triangle \cup \bigcup _{(a,b) \in C} \{\triangle ^{ab}\}$ and $G'$ is the graph defined by

$$\begin{aligned} V(G')&= V(G) \cup \bigcup _{(a,b) \in C} \big (\{\triangle ^{ab}, a^{ab}, b^{ab}\} {}\cup Y^{ab}_{a{\Circle }} \cup Y^{ab}_{b{\Circle }} \cup Y^{ab}_{a\square } \cup Y^{ab}_{b\square }\\&\quad \, {}\cup Z^{ab}_{a{\Circle }} \cup Z^{ab}_{b{\Circle }} \cup Z^{ab}_{a\square } \cup Z^{ab}_{b\square }\big ),\\ E(G')&= E(G) \cup \bigcup _{(a,b) \in C} \bigcup _{x \in \{a,b\}} \big ( \{(\triangle ^{ab}, x^{ab})\} \cup (\{x\} \times Y^{ab}_{x{\Circle }}) \cup (\{x^{ab}\} \times Z^{ab}_{x{\Circle }})\\&\quad \, {}\cup {} \bigcup _{1\le i \le n+3} x_i^{ab} \oplus x_{i+1}^{ab} \big ). \end{aligned}$$

We illustrate our construction in Fig. 5.

#### Lemma 2

Let $I = (G,k,V_\square ,V_\triangle ,C)$ be a ${\textsc {Secure}\,\textsc {Set}^\textsc {FNC}}$ instance, let *A* be the set of solutions of *I* and let *B* be the set of solutions of $\tau ^\text {FNC}(I)$. There is a bijection $g: A \rightarrow B$ such that ${|g(S) |} = \sigma _{I}^\text {FNC}({|S |})$ holds for every $S \in A$.

#### Proof

We use the same auxiliary notation as in Definition 5 and we define *g* as $S \mapsto S \cup \bigcup _{(a,b) \in C,\, x \in S \cap \{a,b\}} (\{\triangle ^{ab}, x^{ab}\} \cup Y^{ab}_{x{\Circle }} \cup Z^{ab}_{x{\Circle }})$. For every $S \in A$, we thus obtain ${|g(S) |} = \sigma _{I}^\text {FNC}({|S |})$, and we first show that indeed $g(S) \in B$.

Let $S \in A$ and let $S'$ denote *g*(*S*). Obviously $S'$ satisfies $V_\square ' \cap S' = \emptyset$ and $V_\triangle ' \subseteq S'$. To see that $S'$ is secure in $G'$, let $X'$ be an arbitrary subset of $S'$. Since *S* is secure in *G* and $X' \cap V(G) \subseteq S$, there is a defense $\mu : N_G[X' \cap V(G)] {\setminus } S \rightarrow N_G[X' \cap V(G)] \cap S$. We now construct a defense $\mu ': N_{G'}[X'] {\setminus } S' \rightarrow N_{G'}[X'] \cap S'$. For any attacker *v* of $X'$ in $G'$, we distinguish three cases.

- If *v* is some $x_i^{ab\square } \in Y^{ab}_{x\square } \cup Z^{ab}_{x\square }$ for some $(a,b) \in C$ and $x \in \{a,b\}$, we set $\mu '(v) = x_i^{ab}$. This element is in $N_{G'}[X']$ since *v* is only adjacent to $x_i^{ab}$ or neighbors of it.
- If *v* is $a^{ab}$ or $b^{ab}$ for some $(a,b) \in C$, its only neighbor in $X'$ can be $\triangle ^{ab}$ and we set $\mu '(v) = \triangle ^{ab}$.
- Otherwise *v* is in $N_G[X' \cap V(G)] {\setminus } S$ (by our construction of $S'$). Since the codomain of $\mu$ is a subset of the codomain of $\mu '$, we may set $\mu '(v) = \mu (v)$.

Since $\mu '$ is injective, each attack on $X'$ in $G'$ can be repelled by $S'$. Hence $S'$ is secure in $G'$.

Clearly *g* is injective. It remains to show that *g* is surjective. Let $S'$ be a solution of $\tau ^\text {FNC}(I)$. First we make the following observations for each $(a,b) \in C$ and each $x \in \{a,b\}$:

- If some $x^{ab}_i \in Y^{ab}_{x{\Circle }}$ is in $S'$, then $Y^{ab}_{x{\Circle }} \cup Z^{ab}_{x{\Circle }} \cup \{x\} \subseteq S'$ by Observation 2.
- If some $x^{ab}_i \in Z^{ab}_{x{\Circle }}$ is in $S'$, then $Y^{ab}_{x{\Circle }} \cup Z^{ab}_{x{\Circle }} \cup \{x^{ab}\} \subseteq S'$ for the same reason.
- If $x \in S'$, then $Y^{ab}_{x{\Circle }} \cap S' \ne \emptyset$. To see this, suppose $x \in S'$. Let $D_x$ consist of those pairs $(c,d) \in C$ such that $x \in \{c,d\}$ and $Y^{cd}_{x{\Circle }} \cap S' \ne \emptyset$, and let $A_x$ consist of those pairs $(c,d) \in C$ such that $x \in \{c,d\}$ and $Y^{cd}_{x{\Circle }} \cap S' = \emptyset$. Now let $X' = \{x\} \cup \{x^{cd}_1,\dots ,x^{cd}_n \mid (c,d) \in D_x\}$. By the previous observations, $X' \subseteq S'$. The defenders of $X'$ are the element *x*, the ${|D_x |} \cdot (n+1)$ elements of $\bigcup _{(c,d) \in D_x} Y^{cd}_{x{\Circle }}$ and perhaps some elements of $N_G(x)$, which consists of at most $n-1$ vertices. The attackers of $X'$ are the ${|D_x |} \cdot (n+1)$ elements of $\bigcup _{(c,d) \in D_x} Y^{cd}_{x\square }$, the ${|A_x |} \cdot (n+1)$ elements of $\bigcup _{(c,d) \in A_x} Y^{cd}_{x{\Circle }}$ and perhaps some elements of $N_G(x)$. Thus, if $A_x$ is nonempty, then the set $X'$ has more attackers than defenders in $G'$. However, $S'$ is secure, so $A_x$ must be empty, which implies $Y^{ab}_{x{\Circle }} \cap S' \ne \emptyset$.
- If $x^{ab} \in S'$, then $Z^{ab}_{x{\Circle }} \cap S' \ne \emptyset$ by Observation 1.

So for each $(a,b) \in C$ and $x \in \{a,b\}$, $S'$ contains either all or none of $\{x, x^{ab}\} \cup Y^{ab}_{x{\Circle }} \cup Z^{ab}_{x{\Circle }}$.

For every $(a,b) \in C$, $S'$ contains $a^{ab}$ or $b^{ab}$, since $\triangle ^{ab} \in S'$, whose neighbors are $a^{ab}$ and $b^{ab}$. It follows that ${|S' |} > {|C |} \cdot (n+6)$ even if $S'$ contains only one of each $(a,b) \in C$. If, for some $(a,b) \in C$, $S'$ contained both *a* and *b*, we could derive a contradiction to ${|S' |} \le \sigma _{I}^\text {FNC}(k) = k + {|C |} \cdot (n+6)$ because then ${|S' |}> ({|C |}+1) \cdot (n+6) > \sigma _{I}^\text {FNC}(k)$. So $S'$ contains either *a* or *b* for any $(a,b) \in C$.

We construct $S = S' \cap V(G)$ and observe that $S' = g(S)$, $V_\triangle \subseteq S$, $V_\square \cap S = \emptyset$, and ${|S \cap \{a,b\} |} = 1$ for each $(a,b) \in C$. It remains to show that *S* is secure in *G*. Let *X* be an arbitrary subset of *S*. We construct $X' = X \cup \bigcup _{(a,b) \in C, x \in X \cap \{a,b\}} Y^{ab}_{x{\Circle }}$ and observe that each $Y^{ab}_{x{\Circle }}$ we put into $X'$ entails ${|Y^{ab}_{x{\Circle }} \cup \{x_{n+2}^{ab}\} |} = n+2$ additional defenders and ${|Y^{ab}_{x\square } \cup \{x_{n+2}^{ab\square }\} |} = n+2$ additional attackers of $X'$ in $G'$ compared to *X* in *G*; so ${|N_{G'}[X'] \cap S' |} - {|N_G[X] \cap S |} = {|N_{G'}[X'] {\setminus } S' |} - {|N_G[X] {\setminus } S |}$. Clearly $X'$ is a subset of $S'$, so ${|N_{G'}[X'] \cap S' |} \ge {|N_{G'}[X'] {\setminus } S' |}$ as $S'$ is secure in $G'$. We conclude ${|N_G[X] \cap S |} \ge {|N_G[X] {\setminus } S |}$. Hence *S* is secure in *G*. $\square$

As $\tau ^\text {FNC}$ is clearly computable in polynomial time, the following result follows:

#### Corollary 1

$${\textsc {Secure}\,\textsc {Set}^\textsc {FN}}$$ is $$\mathrm {\Sigma ^P_2}$$-hard.

The instances of ${\textsc {Secure}\,\textsc {Set}^\textsc {FNC}}$ are identical to the instances of the exact variant, so $\tau ^\text {FNC}$ is also applicable to the exact case. In fact it turns out that this gives us also a reduction from ${\textsc {Exact}\,\textsc {Secure}\,\textsc {Set}^\textsc {FNC}}$ to ${\textsc {Exact}\,\textsc {Secure}\,\textsc {Set}^\textsc {FN}}$.

#### Corollary 2

$${\textsc {Exact}\,\textsc {Secure}\,\textsc {Set}^\textsc {FN}}$$ is $$\mathrm {\Sigma ^P_2}$$-hard.

#### Proof

Let *I* and $I' = \tau ^\text {FNC}(I)$ be our ${\textsc {Exact}\,\textsc {Secure}\,\textsc {Set}^\textsc {FNC}}$ and ${\textsc {Exact}\,\textsc {Secure}\,\textsc {Set}^\textsc {FN}}$ instances, respectively, and let *k* and $k'$ denote their respective solution sizes. By Lemma 2, there is a bijection *g* between the solutions of *I* and the solutions of $I'$ such that, for every solution *S* of *I*, *g*(*S*) has $\sigma _{I}^\text {FNC}(k) = k'$ elements, and for every solution $S'$ of $I'$, $g^{-1}(S')$ has *k* elements since $\sigma _{I}^\text {FNC}$ is invertible. $\square$

### Hardness of Secure Set with Forbidden Vertices

Now we present a transformation $\tau ^\text {FN}$ that eliminates necessary vertices. Our transformation not only operates on a problem instance, but also requires an ordering $\preceq$ of the necessary vertices. For now, we can consider this as an arbitrary ordering. It will become more important in Sect. 4.1, where we reuse this transformation for showing $\mathrm {W[1]}$-hardness w.r.t. treewidth.

**Fig. 6:**
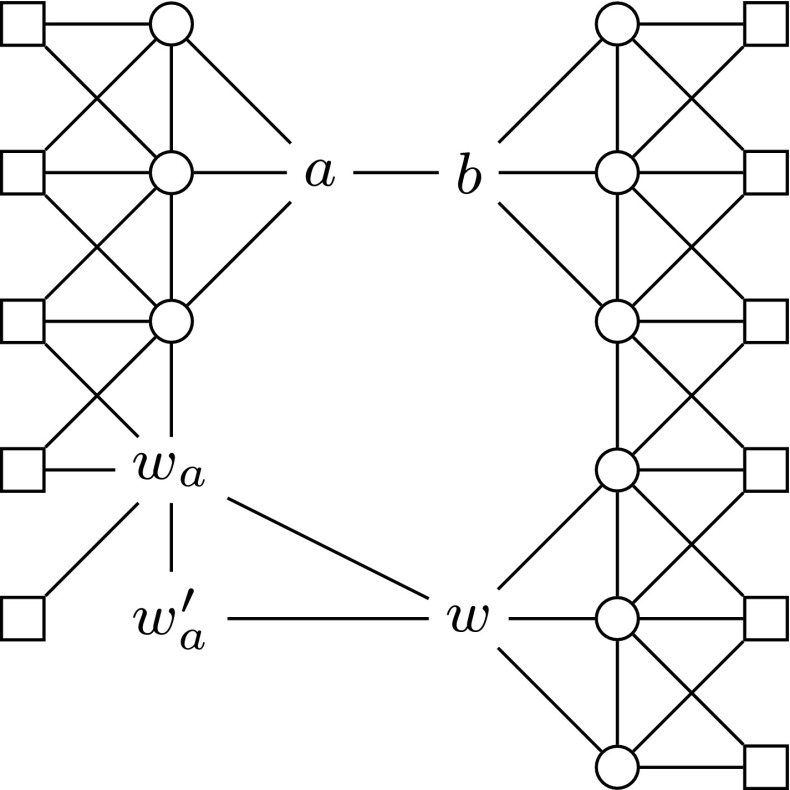
Result of the transformation $\tau ^\text {FN}{}$ applied to an example graph with two adjacent vertices *a* and *b*, where *b* is necessary. Every solution in the depicted graph contains *b*, *w* and $w_a'$

Before formally defining the transformation $\tau ^\text {FN}$, we refer to Fig. 6, which shows the result for a simple example graph with only two vertices *a* and *b*, of which *b* is necessary. The basic idea is that the vertex *w* must be in every solution *S* because any vertex that is in *S* also eventually forces *w* to be in *S*. Once $w \in S$, the construction to the right of *w* makes sure that $b \in S$.

#### Definition 6

We define a function $\tau ^\text {FN}$, which assigns a ${\textsc {Secure}\,\textsc {Set}^\textsc {F}}$ instance to each pair $(I,{\preceq })$, where $I = (G,k,V_\square ,V_\triangle )$ is a ${\textsc {Secure}\,\textsc {Set}^\textsc {FN}}$ instance and ${\preceq }$ is an ordering of the elements of $V_\triangle$. For this, let $V_{\Circle }$ denote $V(G) {\setminus } (V_\square \cup V_\triangle )$. We use *n* to denote ${|V(G) |}$, and we first define a function $\sigma _{I}^\text {FN} : x \mapsto xn + 3x + n - {|V_\triangle |} + {|V_{\Circle } |} + 2$. We use *W* to denote the set of new vertices $\{w\} \cup \{w_v, w_v', w_v^\square , w_v'^\square \mid v \in V_{\Circle }\}$. The intention is for each $w_v^\square$ and $w_v'^\square$ to be forbidden, for *w* and each $w_v'$ to be in every secure set, and for $w_v$ to be in a secure set iff *v* is in it at the same time. We write $V^+$ to denote $V_\triangle \cup V_{\Circle }\cup \{w\}$; for each $v \in V^+$, we use $A_v$ to denote the set of new vertices $\{v_1, \dots , v_{n+1}, v_1^\square , \dots , v_{n+1}^\square \}$, and we use shorthand notation $A_v^{\Circle }= \{v_1, \dots , v_{n+1}\}$ and $A_v^\square = \{v_1^\square , \dots , v_{n+1}^\square \}$. The intention is for each $v_i^\square$ to be forbidden and for each $v_i$ to be in a secure set iff *v* is in it at the same time. We use the notation $u \oplus v$ to denote the set of edges $\{(u,v), (u,u^\square ), (v,v^\square ), (u,v^\square ), (v,u^\square )\}$. If $V_\triangle = \emptyset$, let $P = \emptyset$; otherwise let *P* be the set consisting of all pairs (*u*, *v*) such that *v* is the direct successor of *u* according to ${\preceq }$, as well as the pair (*u*, *w*), where *u* is the greatest element according to ${\preceq }$. Now we define $\tau ^\text {FN}(I,{\preceq }) = (G',k',V_\square ')$, where $V'_\square = V_\square \cup \{w_v^\square , w_v'^\square \mid v \in V_{\Circle }\} \cup \bigcup _{v \in V^+} A_v^\square$, $k' = \sigma _{I}^\text {FN}(k)$, and $G'$ is the graph defined by

$$\begin{aligned} V(G') ={}&V(G) \cup W \cup \bigcup _{v \in V^+} A_v,\\ E(G') ={}&E(G) \cup \{ (v, v_i) \mid v \in V^+,\; 1 \le i \le n+1 \}\\&{}\cup {} \bigcup _{v \in V^+,\; 1 \le i \le n} v_i \oplus v_{i+1} \cup \bigcup _{(u,v) \in P} u_{n+1} \oplus v_1\\&{}\cup {} \bigcup _{v \in V_{\Circle }} v_{n+1} \oplus w_v \cup \left\{ (w,w_v), (w,w_v'), (w_v,w_v'), (w_v,w_v'^\square ) \mid v \in V_{\Circle }\right\} . \end{aligned}$$

**Fig. 7:**
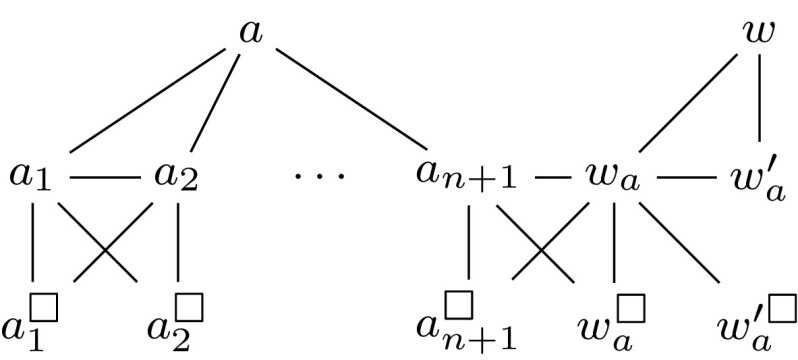
Illustration of the gadget that makes sure *w* and $w_a'$ are in every secure set. The vertex *a* is a non-necessary, non-forbidden vertex from the ${\textsc {Secure}\,\textsc {Set}^\textsc {FN}}$ instance and may have other neighbors from this instance. The vertex *w* has two neighbors (as depicted here) for each non-necessary, non-forbidden vertex from the ${\textsc {Secure}\,\textsc {Set}^\textsc {FN}}$ instance, and additionally the neighbors depicted in Fig. 8

**Fig. 8:**
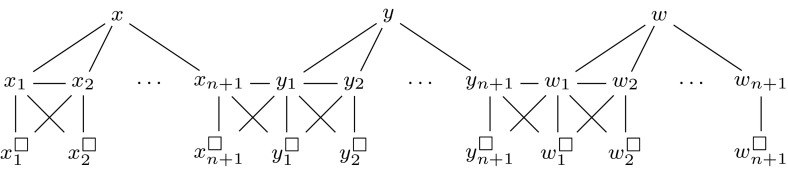
Illustration of the gadget that makes sure every secure set contains all necessary vertices as it must contain *w*. Here we assume there are the two necessary vertices *x* and *y*, and we use the ordering $x \preceq y$

We illustrate our construction in Figs. 7 and 8.

We now prove that $\tau ^\text {FN}$ yields a correct reduction for any ordering $\preceq$.

#### Lemma 3

Let $I = (G,k,V_\square ,V_\triangle )$ be a ${\textsc {Secure}\,\textsc {Set}^\textsc {FN}}$ instance, let ${\preceq }$ be an ordering of $V_\triangle$, let *A* be the set of solutions of *I* and let *B* be the set of solutions of $\tau ^\text {FN}(I,{\preceq })$. There is a bijection $g: A \rightarrow B$ such that ${|g(S) |} = \sigma _{I}^\text {FN}({|S |})$ holds for every $S \in A$.

#### Proof

We use the same auxiliary notation as in Definition 6 and we define *g* as $S \mapsto S \cup \bigcup _{v \in S} A_v^{\Circle }\cup A_w^{\Circle }\cup \{w\} \cup \{w_v' \mid v \in V_{\Circle }\} \cup \{w_v \mid v \in S \cap V_{\Circle }\}$. For every $S \in A$, we thus obtain ${|g(S) |} = {|S |} + {|S |} (n+1) + (n+1) + 1 + {|V_{\Circle } |} + ({|S |}-{|V_\triangle |}) = \sigma _{I}^\text {FN}({|S |})$, and we first show that indeed $g(S) \in B$.

Let $S \in A$ and let $S'$ denote *g*(*S*). Obviously $S'$ satisfies $V_\square ' \cap S' = \emptyset$. To see that $S'$ is secure in $G'$, let $X'$ be an arbitrary subset of $S'$. Since *S* is secure in *G* and $X' \cap V(G) \subseteq S$, there is a defense $\mu : N_G[X' \cap V(G)] {\setminus } S \rightarrow N_G[X' \cap V(G)] \cap S$. We now construct a defense $\mu ': N_{G'}[X'] {\setminus } S' \rightarrow N_{G'}[X'] \cap S'$. For any attacker *a* of $X'$ in $G'$, we distinguish the following cases:

- If *a* is some $v_i^\square \in A_v^\square$ for some $v \in V^+$, then *a* can only attack either $v_i$ or a neighbor of $v_i$, all of which are in $S'$, and we set $\mu '(a) = v_i$.
- Similarly, if *a* is $w_v^\square$ for some $v \in V_{\Circle }$, then we set $\mu '(a) = w_v$.
- If *a* is $w_v'^\square$ for some $v \in V_{\Circle }$, then *a* attacks $w_v$ and we set $\mu '(a) = w_v'$.
- If *a* is $w_v$ for some $v \in V_{\Circle }$, then it attacks *w* or $w_v'$, which is not used for repelling any attack because $w_v'^\square$ cannot attack $X'$, so we set $\mu '(a) = w_v'$.
- Otherwise *a* is in $N_G[X' \cap V(G)] {\setminus } S$ (by our construction of $S'$). Since the codomain of $\mu$ is a subset of the codomain of $\mu '$, we may set $\mu '(a) = \mu (a)$.

Since $\mu '$ is injective, each attack on $X'$ in $G'$ can be repelled by $S'$. Hence $S'$ is secure in $G'$.

Clearly *g* is injective. It remains to show that *g* is surjective. Let $S'$ be a solution of $\tau ^\text {FN}(I,{\preceq })$. We first show that $V_\triangle \cup \{w\} \subseteq S'$:

- If $S'$ contains some $v \in V_\triangle \cup V_{\Circle }$, then $S'$ contains an element of $A_v^{\Circle }$ by Observation 1.
- If $S'$ contains an element of $A_v^{\Circle }$ for some $v \in V^+$, then $\{v\} \cup A_v^{\Circle }\subseteq S'$ by Observation 2.
- If $v_{n+1} \in S'$ for some $v \in V_{\Circle }$, then $w_v \in S'$ for the same reason.
- Furthermore, if $S'$ contains an element of $A_v^{\Circle }$ for some $v \in V_\triangle \cup \{w\}$, then also $A_{u}^{\Circle }\subseteq S'$ for every $u \in V_\triangle \cup \{w\}$ for the same reason.
- If $w_v \in S'$ for some $v \in V_{\Circle }$, then $\{w,w_v',v_{n+1}\} \subseteq S'$ by Observation 2.
- If $w_v' \in S'$ for some $v \in V_{\Circle }$, then $w \in S'$ because at least $w_v$ or *w* must be in $S'$ and the former implies $w \in S'$ as we have seen.
- The previous observations show that any vertex being in $S'$ implies $w \in S'$. Since $S'$ is nonempty, it follows that $w \in S'$. We now show that $S'$ contains an element of $A_w^{\Circle }$. Suppose the contrary, let $U = S' \cap \{w_v \mid v \in V_{\Circle }\}$, let $U' = S' \cap \{w_v' \mid v \in V_{\Circle }\}$ and consider $X' = \{w\} \cup U$. The defenders of $X'$ consist of exactly $1 + {|U' |} + 2 {|U |}$ elements, whereas there are exactly $(n+1) + ({|V_{\Circle } |}- {|U' |}) + ({|V_{\Circle } |} - {|U |}) + 3 {|U |}$ attackers. With ${|V_{\Circle } |} \ge {|U' |} \ge {|U |}$ and $n > 0$ in mind, we arrive at the contradiction ${|N_{G'}[X'] \cap S' |} < {|N_{G'}[X'] {\setminus } S' |}$.
- The previous observations show that for every $v \in V_\triangle \cup \{w\}$ it holds that $\{v\} \cup A_v^{\Circle }\subseteq S'$. Finally, we show that $\{w_v' \mid v \in V_{\Circle }\} \subseteq S'$. Suppose, for the sake of contradiction, that there is some $u \in V_{\Circle }$ such that $w_u' \notin S'$. We have seen that the latter can only be the case if $u \notin S'$. Observe that $\{w\} \cup \{w_i \mid 2 \le i \le n+1\} \cup \{w_v \mid v \in V_{\Circle }\cap S'\}$ is a subset of $S'$ that is attacked by $\{w_u'\} \cup A_w^\square \cup \{v_{n+1}^\square , w_v^\square , w_v'^\square \mid v \in V_{\Circle }\cap S'\} \cup \{w_v \mid v \in V_{\Circle }{\setminus } S'\}$, but the defenders are a proper subset of $\{w\} \cup A_w^{\Circle }\cup \{v_{n+1}, w_v, w_v' \mid v \in V_{\Circle }\cap S'\} \cup \{w_v' \mid v \in V_{\Circle }{\setminus } S'\}$. This contradicts $S'$ being secure in $G'$.

Let $S = S' \cap V(G)$. By the previous observations, it is easy to see that $S' = g(S)$. It remains to show that *S* is secure in *G*. Let *X* be an arbitrary subset of *S*. We construct $X' = X \cup \bigcup _{v \in X} A^{\Circle }_v$ and observe that the number of additional defenders of $X'$ in $G'$ compared to *X* in *G* is equal to the number of additional attackers; formally ${|N_{G'}[X'] \cap S' |} - {|N_G[X] \cap S |} = {|N_{G'}[X'] {\setminus } S' |} - {|N_G[X] {\setminus } S |}$. Clearly $X' \subseteq S'$, so ${|N_{G'}[X'] \cap S' |} \ge {|N_{G'}[X'] {\setminus } S' |}$ as $S'$ is secure in $G'$. Consequently ${|N_G[X] \cap S |} \ge {|N_G[X] {\setminus } S |}$. Hence *S* is secure in *G*. $\square$

Given an ordering ${\preceq }$, clearly $\tau ^\text {FN}(I,\preceq )$ is computable in polynomial time. We can thus easily obtain a reduction from ${\textsc {Secure}\,\textsc {Set}^\textsc {FN}}$ to ${\textsc {Secure}\,\textsc {Set}^\textsc {F}}$ by first computing an arbitrary ordering ${\preceq }$ of the necessary vertices in polynomial time. This also gives us a hardness result for the exact case, analogous to Corollary 2.

#### Corollary 3

${\textsc {Secure}\,\textsc {Set}^\textsc {F}}$ and ${\textsc {Exact}\,\textsc {Secure}\,\textsc {Set}^\textsc {F}}$ are $\mathrm {\Sigma ^P_2}$-hard.

### Hardness of Secure Set

We now introduce a transformation $\tau ^\text {F}$ that eliminates forbidden vertices. The basic idea is that we ensure that a forbidden vertex *f* is never part of a solution by adding so many neighbors to *f* that we could only defend *f* by exceeding the bound on the solution size.

#### Definition 7

We define a function $\tau ^\text {F}$, which assigns a Secure Set instance to each ${\textsc {Secure}\,\textsc {Set}^\textsc {F}}$ instance $I = (G,k,V_\square )$. For each $f \in V_\square$, we introduce new vertices $f', f_1, \dots , f_{2k}$. Now we define $\tau ^\text {F}(I) = (G',k)$, where $G'$ is the graph defined by

$$\begin{aligned} V(G') ={}&V(G) \cup \{f', f_1, \dots , f_{2k} \mid f \in V_\square \},\\ E(G') ={}&E(G) \cup \{ (f,f_i),\; (f',f_i) \mid f \in V_\square ,\; 1 \le i \le 2k\}. \end{aligned}$$

#### Lemma 4

Every ${\textsc {Secure}\,\textsc {Set}^\textsc {F}}$ instance *I* has the same solutions as the Secure Set instance $\tau ^\text {F}(I)$.

#### Proof

Let $I = (G,k,V_\square )$ and $\tau ^\text {F}(I) = (G',k)$. Each secure set *S* in *G* is also secure in $G'$ because the subgraph of *G* induced by $N_G[S]$ is equal to the subgraph of $G'$ induced by $N_{G'}[S]$. Now let $S'$ be a solution of $\tau ^\text {F}(I)$. For every $f \in V_\square$, neither *f* nor $f'$ are in $S'$ because each of these vertices has at least 2*k* neighbors, and $S'$ cannot contain any $f_i$ because $N_{G'}(f_i) = \{f,f'\}$. Hence $S'$ is also secure in *G* as the subgraphs induced by the respective neighborhoods are again equal. $\square$

This immediately yields the following result.

#### Corollary 4

Secure Set and Exact Secure Set are $\mathrm {\Sigma ^P_2}$-hard.

## Complexity of Secure Set Parameterized by Treewidth

In this section we study the parameterized complexity of the Secure Set problem when treewidth is the parameter.

We first show that all variants of Secure Set considered in this paper are $\mathrm {W[1]}$-hard for this parameter by reusing some reductions from Sect. 3 and proving that they preserve bounded treewidth. Under the widely held assumption that $\mathrm {FPT}\ne \mathrm {W[1]}$, this rules out fixed-parameter tractable algorithms for these problems.

Second, we show that the $\mathrm {\text {co-}NP}$-complete Secure Set Verification problem is solvable in linear time on instances whose treewidth is bounded by a constant. We do this by providing a fixed-parameter linear algorithm that performs dynamic programming on a tree decomposition of the input graph. Although bounded treewidth most likely does not lead to fixed-parameter tractability of the problem of *finding* secure sets, this proves that it does for the problem of verifying whether a given set is secure.

Third, we show that all the variants of the Secure Set problem considered in this paper are solvable in polynomial time on instances whose treewidth is bounded by a constant. We again do this by providing a polynomial-time dynamic programming algorithm, but this time the degree of the polynomial depends on the treewidth.

### Hardness of Secure Set Parameterized by Treewidth

In this subsection, we prove the following theorem:

#### Theorem 2

The following problems are all $\mathrm {W[1]}$-hard when parameterized by treewidth: Secure Set, Exact Secure Set, ${\textsc {Secure}\,\textsc {Set}^\textsc {F}}$, ${\textsc {Exact}\,\textsc {Secure}\,\textsc {Set}^\textsc {F}}$, ${\textsc {Secure}\,\textsc {Set}^\textsc {FN}}$, ${\textsc {Exact}\,\textsc {Secure}\,\textsc {Set}^\textsc {FN}}$, ${\textsc {Secure}\,\textsc {Set}^\textsc {FNC}}$, and ${\textsc {Exact}\,\textsc {Secure}\,\textsc {Set}^\textsc {FNC}}$.

To prove this, we reduce from the following problem [3], which is known to be $\mathrm {W[1]}$-hard [29] parameterized by the treewidth of the graph: 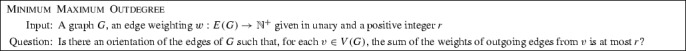


#### Lemma 5

${\textsc {Secure}\,\textsc {Set}^\textsc {FNC}}$ and ${\textsc {Exact}\,\textsc {Secure}\,\textsc {Set}^\textsc {FNC}}$, both parameterized by the treewidth of the primal graph, are $\mathrm {W[1]}$-hard.

#### Proof

**Fig. 9:**
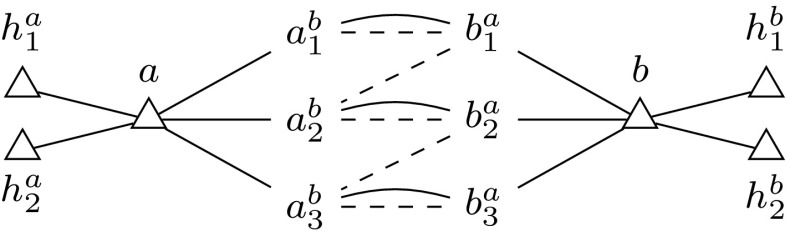
Result of our transformation on a sample Minimum Maximum Outdegree instance with $r=3$ and two vertices *a*, *b* that are connected by an edge of weight 3. Complementary vertex pairs are shown via *dashed lines*. Necessary vertices have a $\triangle$ symbol next to their name

Let an instance of Minimum Maximum Outdegree be given by a graph *G*, an edge weighting $w: E(G) \rightarrow \mathbb {N}^+$ in unary and a positive integer *r*. From this we construct an instance of both ${\textsc {Secure}\,\textsc {Set}^\textsc {FNC}}$ and ${\textsc {Exact}\,\textsc {Secure}\,\textsc {Set}^\textsc {FNC}}$. An example is given in Fig. 9. For each $v \in V(G)$, we define the set of new vertices $H_v = \{h^v_1, \dots , h^v_{r-1}\}$, and for each $(u,v) \in E(G)$, we define the sets of new vertices $V_{uv} = \{u^v_1, \dots , u^v_{w(u,v)}\}$ and $V_{vu} = \{v^u_1, \dots , v^u_{w(u,v)}\}$. We now define the graph $G'$ with

$$\begin{aligned} V(G') ={}&V(G) \cup \bigcup _{v \in V(G)} H_v \cup \bigcup _{(u,v) \in E(G)} (V_{uv} \cup V_{vu}),\\ E(G') ={}&\{(v,h) \mid v \in V(G),\; h \in H_v\}\\&{}\cup {}\{(u,x) \mid (u,v) \in E(G),\; x \in V_{uv}\}\\&{}\cup {} \{(x,v) \mid (u,v) \in E(G),\; x \in V_{vu}\}\\&{}\cup {}\left\{ \left( u^v_i,v^u_i\right) \mid (u,v) \in E(G),\; 1 \le i \le w(u,v)\right\} . \end{aligned}$$

We also define the set of complementary vertex pairs $C = \{(u^v_i,v^u_i) \mid (u,v) \in E(G),\; 1 \le i \le w(u,v)\} \cup \{(v^u_i,u^v_{i+1}) \mid (u,v) \in E(G),\; 1 \le i < w(u,v)\}$. Finally, we define the set of necessary vertices $V_\triangle = V(G) \cup \bigcup _{v \in V(G)} H_v$ and $k = {|V_\triangle |} + \sum _{(u,v) \in E(G)} w(u,v)$. We use *I* to denote $(G',k,C,V_\triangle ,\emptyset )$, which is an instance of ${\textsc {Secure}\,\textsc {Set}^\textsc {FNC}}$ and also of ${\textsc {Exact}\,\textsc {Secure}\,\textsc {Set}^\textsc {FNC}}$. Obviously *I* is a positive instance of ${\textsc {Secure}\,\textsc {Set}^\textsc {FNC}}$ iff it is a positive instance of ${\textsc {Exact}\,\textsc {Secure}\,\textsc {Set}^\textsc {FNC}}$ because the necessary and complementary vertices make sure that every solution of the ${\textsc {Secure}\,\textsc {Set}^\textsc {FNC}}$ instance *I* has exactly *k* elements. Hence we only consider ${\textsc {Secure}\,\textsc {Set}^\textsc {FNC}}$.

The intention is that for each orientation of *G* we have a solution candidate *S* in *I* such that an edge orientation from *u* to *v* entails $V_{vu} \subseteq S$ and $V_{uv} \cap S = \emptyset$, and the other orientation entails $V_{uv} \subseteq S$ and $V_{vu} \cap S = \emptyset$. For each outgoing edge of *v* in the orientation of *G*, there are as many attackers of *v* in *I* as the weight of that edge. Together with $H_v$, *v* can repel up to *r* such attacks. The other neighbors of *v* that are in *S* cannot help *v* since they are in turn attacked by their neighbors.

Clearly *I* can be computed in polynomial time. We now show that the treewidth of the primal graph of *I* depends only on the treewidth of *G*. We do so by modifying an optimal tree decomposition $\mathcal {T}$ of *G* as follows:

1. For each $(u,v) \in E(G)$, we take an arbitrary node whose bag *B* contains both *u* and *v* and add to its children a chain of nodes $N_1, \dots , N_{w(u,v)-1}$ such that the bag of $N_i$ is $B \cup \{u^v_i, u^v_{i+1}, v^u_i, v^u_{i+1}\}$.
2. For each $v \in V(G)$, we take an arbitrary node whose bag *B* contains *v* and add to its children a chain of nodes $N_1, \dots , N_{r-1}$ such that the bag of $N_i$ is $B \cup \{h^v_i\}$.

It is easy to verify that the result is a valid tree decomposition of the primal graph of *I* and its width is at most the treewidth of *G* plus four.

We claim that (*G*, *w*, *r*) is a positive instance of Minimum Maximum Outdegree iff *I* is a positive instance of ${\textsc {Secure}\,\textsc {Set}^\textsc {FNC}}$.

“*Only if*” *direction* Let *D* be the directed graph given by an orientation of the edges of *G* such that for each vertex the sum of weights of outgoing edges is at most *r*. The set $S = V_\triangle \cup \{v^u_1, \dots , v^u_{w(u,v)} \mid (u,v) \in E(D)\}$ is secure in *G*: Let *X* be an arbitrary subset of *S*. Every attacker must be some element $u^v_i$. If $v^u_i \in X$, then we can use $v^u_i$ to repel the attack from $u^v_i$. Otherwise $u \in X$, so we can use either *u* or one of the $r-1$ elements of $H_u$ to repel the attack from $u^v_i$. These are sufficiently many defenders: For every vertex $v \in V(G) \cap X$, at most *r* neighbors attack *v* as otherwise the sum of weights of outgoing edges of *v* in *D* would be greater than *r*. Finally, it is easy to verify that ${|S |} = k$, $V_\triangle \subseteq S$, and exactly one element of each pair of complementary vertices is in *S*.

“*If*” *direction* Let *S* be a solution of *I*. For every $(u,v) \in E(G)$, either $V_{uv} \subseteq S$ or $V_{vu} \subseteq S$ due to the complementary vertex pairs. We define a directed graph *D* by $V(D) = V(G)$ and $E(D) = \{(u,v) \mid V_{vu} \subseteq S\} \cup \{(v,u) \mid V_{uv} \subseteq S\}$. Suppose there is a vertex *v* in *D* whose sum of weights of outgoing edges is greater than *r*. We construct $X = \{v\} \cup \bigcup _{(u,v) \in E(D)} V_{vu}$, which is a subset of *S*. Now *v* has more than *r* attacking neighbors, but all defenders except *v* and $H_v$ must already defend themselves against their attacking neighbor. This contradicts *S* being secure. $\square$

Now we reduce from ${\textsc {Secure}\,\textsc {Set}^\textsc {FNC}}$ to ${\textsc {Secure}\,\textsc {Set}^\textsc {FN}}$ to show $\mathrm {W[1]}$-hardness of the latter problem. We reuse the function $\tau ^\text {FNC}$ from Definition 5 and show that this gives us a reduction that preserves bounded treewidth.

#### Lemma 6

${\textsc {Secure}\,\textsc {Set}^\textsc {FN}}$, parameterized by the treewidth of the graph, is $\mathrm {W[1]}$-hard.

#### Proof

Let *I* be a ${\textsc {Secure}\,\textsc {Set}^\textsc {FNC}}$ instance whose primal graph we denote by *G*. We obtain an equivalent ${\textsc {Secure}\,\textsc {Set}^\textsc {FN}}$ instance $\tau ^\text {FNC}(I)$, whose graph we denote by $G'$. This reduction is correct, as shown in Lemma 2. It remains to show that the treewidth of $G'$ is bounded by a function of the treewidth of *G*. Let $\mathcal {T}$ be an optimal nice tree decomposition of *G*. We build a tree decomposition $\mathcal {T}'$ of $G'$ by modifying a copy of $\mathcal {T}$ in the following way: For every pair (*a*, *b*) of complementary vertices, we pick an arbitrary node *t* in $\mathcal {T}$ whose bag *B* contains both *a* and *b*, and we add a chain of nodes $N_1, \dots , N_{2n+3}$ between *t* and its parent such that, for $1 \le i \le n+1$, the bag of $N_i$ is $B \cup \{a_i^{ab}, a_i^{ab\square }, a_{i+1}^{ab}, a_{i+1}^{ab\square }\}$, the bag of $N_{n+2}$ is $B \cup \{a^{ab}, b^{ab}, \triangle ^{ab}\} \cup Z_{a{\Circle }}^{ab} \cup Z_{a\square }^{ab} \cup Z_{b{\Circle }}^{ab} \cup Z_{b\square }^{ab}$, and the bag of $N_{n+2+i}$ is $B \cup \{b_{n+3-i}^{ab}, b_{n+3-1}^{ab\square }, b_{n+2-i}^{ab}, b_{n+2-i}^{ab\square }\}$. It is easy to verify that $\mathcal {T}'$ is a valid tree decomposition of $G'$. Furthermore, the width of $\mathcal {T}'$ is at most the width of $\mathcal {T}$ plus 15.

Just like before, we get an analogous result for the exact variant. It can be proved in the same way as Corollary 2.

#### Corollary 5

${\textsc {Exact}\,\textsc {Secure}\,\textsc {Set}^\textsc {FN}}$, parameterized by the treewidth of the graph, is $\mathrm {W[1]}$-hard.

We next show $\mathrm {W[1]}$-hardness of ${\textsc {Secure}\,\textsc {Set}^\textsc {F}}$ by reducing from ${\textsc {Secure}\,\textsc {Set}^\textsc {FN}}$ using the function $\tau ^\text {FN}$ from Definition 6. This function maps a ${\textsc {Secure}\,\textsc {Set}^\textsc {FN}}$ instance, together with an ordering $\preceq$ of the necessary vertices, to an equivalent ${\textsc {Secure}\,\textsc {Set}^\textsc {F}}$ instance. We show that by choosing $\preceq$ appropriately, this gives us a reduction that preserves bounded treewidth.

#### Lemma 7

${\textsc {Secure}\,\textsc {Set}^\textsc {F}}$, parameterized by the treewidth of the graph, is $\mathrm {W[1]}$-hard.

#### Proof

Let $I = (G,k,V_\square ,V_\triangle )$ be a ${\textsc {Secure}\,\textsc {Set}^\textsc {FN}}$ instance and let $\mathcal {T}$ be an optimal nice tree decomposition of *G*. We can compute such a tree decomposition in FPT time [6]. Let ${\preceq }$ be the ordering of the elements of $V_\triangle$ that is obtained in linear time by doing a post-order traversal of $\mathcal {T}$ and sequentially recording the elements that occur for the last time in the current bag. We obtain the ${\textsc {Secure}\,\textsc {Set}^\textsc {F}}$ instance $\tau ^\text {FN}(I,\preceq )$, whose graph we denote by $G'$. This reduction is correct, as shown in Lemma 3, and computable in FPT time. It remains to show that the treewidth of $G'$ is bounded by a function of the treewidth of *G*. To this end, we use $\mathcal {T}$ to build a tree decomposition $\mathcal {T}'$ of $G'$. We initially set $\mathcal {T}' := \mathcal {T}$ and modify it by the following steps:

1. We insert *w* into every bag.
2. For every $(u,v) \in P$, we add *v*, $v_1$ and $v_1^\square$ into the bag of every node between (and including) $t_u^{\mathcal {T}'}$ and $t_v^{\mathcal {T}'}$. Note that the bag of $t_u^{\mathcal {T}'}$ contains both *u* and *v*. After this step, we have increased the bag size of each node by at most five.
3. For each $v \in V^+$, we use $B_v$ to denote the bag of $t_v^{\mathcal {T}'}$ and replace $t_v^{\mathcal {T}'}$ by a chain of nodes $N_1, \dots , N_n$, where $N_n$ is the topmost node and the bag of $N_i$ is $B_v \cup \{v_i, v_i^\square , v_{i+1}, v_{i+1}^\square \}$. After this step, note that, for each $(u,v) \in P$, the bag of the new node $t_u^{\mathcal {T}'}$ contains $u_{n+1}$, $u_{n+1}^\square$, $v_1$ and $v_1^\square$.
4. For each $v \in V_{\Circle }$, we add $w_v$, $w_v^\square$, $w_v'$ and $w_v'^\square$ to the bag of $t_v^{\mathcal {T}'}$, which already contains *w*, $v_{n+1}$, $v_{n+1}^\square$.

It is easy to verify that $\mathcal {T}'$ is a valid tree decomposition of $G'$. Furthermore, the width of $\mathcal {T}'$ is at most the width of $\mathcal {T}$ plus twelve. $\square$

We again get an analogous result for the exact variant.

#### Corollary 6

${\textsc {Exact}\,\textsc {Secure}\,\textsc {Set}^\textsc {F}}$, parameterized by the treewidth of the graph, is $\mathrm {W[1]}$-hard.

Finally, we show $\mathrm {W[1]}$-hardness of Secure Set by reducing from ${\textsc {Secure}\,\textsc {Set}^\textsc {F}}$ while preserving bounded treewidth.

#### Lemma 8

Secure Set, parameterized by the treewidth of the graph, is $\mathrm {W[1]}$-hard.

#### Proof

Let $I = (G,k,V_\square )$ be a ${\textsc {Secure}\,\textsc {Set}^\textsc {F}}$ instance, let $G'$ denote the graph of $\tau ^\text {F}(I)$ and let $\mathcal {T}$ be an optimal nice tree decomposition of *G*. We build a tree decomposition $\mathcal {T}'$ of $G'$ by modifying a copy of $\mathcal {T}$ in the following way: For every $f \in V_\square$, we pick an arbitrary node *t* in $\mathcal {T}$ whose bag *B* contains *f*, and we add a chain of nodes $N_1, \dots , N_{2k}$ between *t* and its parent such that, for $1 \le i \le 2k$, the bag of $N_i$ is $B \cup \{f', f_i\}$. It is easy to verify that $\mathcal {T}'$ is a valid tree decomposition of $G'$. Furthermore, the width of $\mathcal {T}'$ is at most the width of $\mathcal {T}$ plus two. $\square$

We again get an analogous result for the exact variant.

#### Corollary 7

Exact Secure Set, parameterized by the treewidth of the input graph, is $\mathrm {W[1]}$-hard.

### A Fixed-Parameter Tractable Algorithm for Secure Set Verification

While we have seen in Sect. 4.1 that Secure Set parameterized by treewidth is most likely not FPT, we now present a positive result: The $\mathrm {\text {co-}NP}$-complete [21] Secure Set Verification problem, which consists of checking whether a given set $\widehat{S}$ is secure in a graph *G*, is FPT parameterized by the treewidth of *G*. We show this by giving a fixed-parameter linear algorithm that follows the principle of dynamic programming on a tree decomposition $\mathcal {T}$ of *G*. The core idea is the following: For each node *t* of $\mathcal {T}$ and each $X \subseteq \widehat{S}\cap \chi (t)$, we store an integer $c_{\widehat{S},t}(X)$, which indicates that *X* can be extended to a set $\widehat{X}\subseteq \widehat{S}$ using “forgotten” vertices from further down in $\mathcal {T}$ in such a way that the difference between defenders and attackers of $\widehat{X}$ is $c_{\widehat{S},t}(X)$ and $\widehat{X}$ is the “worst” subset of $\widehat{S}$ that can be obtained in this way. To compute these values, we traverse $\mathcal {T}$ from the bottom up and use recurrence relations to compute the values for the current node *t* of $\mathcal {T}$ based on the values we have computed for the children of *t*. If we then look at the values we have computed at the root of $\mathcal {T}$, we can decide if there is a subset of $\widehat{S}$ that is “bad enough” to witness that $\widehat{S}$ is not secure.

Dynamic programming algorithms like this are quite common for showing $\mathrm {FPT}$ membership w.r.t. treewidth and some examples can be found in [13, 26]. Proving their correctness is a usually rather tedious structural induction argument along the tree decomposition: At every node *t* of $\mathcal {T}$, we have to prove that the recurrence relations indeed characterize the value they are supposed to represent. Examples of such proofs can be found in [13].

We now formally define the values that we will compute at each tree decomposition node. Let *G* be a graph with a nice tree decomposition $\mathcal {T}$ and let $\widehat{S}\subseteq V(G)$ be the candidate for which we want to check if it is secure. For each node *t* of $\mathcal {T}$ and each set of vertices *A*, we define $A_t = \{a \in A \mid a \in \chi (t'),\, t' \text { is a descendant of } t\}$. For any $\widehat{X}\subseteq \widehat{S}$, we call ${|N_G[\widehat{X}]_t \cap \widehat{S} |} - {|N_G[\widehat{X}]_t {\setminus } \widehat{S} |}$ the *score* of $\widehat{X}$ w.r.t. $\widehat{S}$ at *t* (or just the score of $\widehat{X}$ if $\widehat{S}$ and *t* are clear from the context) and denote it by ${\text {score}}_{\widehat{S},t}(\widehat{X})$. Furthermore, we call ${|N_G[\widehat{X}] \cap \chi (t) \cap \widehat{S} |} - {|(N_G[\widehat{X}] \cap \chi (t)) {\setminus } \widehat{S} |}$ the *local score* of $\widehat{X}$ w.r.t. $\widehat{S}$ at *t* and denote it by ${\text {lscore}}_{\widehat{S},t}(\widehat{X})$. Finally, for each $X \subseteq \widehat{S}\cap \chi (t)$, we define the value

$$\begin{aligned} c_{\widehat{S},t}(X)= \min _{\widehat{X}\subseteq \widehat{S}_t,\; \widehat{X}\cap \chi (t) = X}\left\{ {\text {score}}_{\widehat{S},t}(\widehat{X})\right\} . \end{aligned}$$

When *r* is the root of $\mathcal {T}$, both $\widehat{S}_r = \widehat{S}$ and $\chi (r) = \emptyset$ hold, so $\widehat{S}$ is secure if and only if $c_{\widehat{S},r}(\emptyset )$ is nonnegative.

We now describe how to compute all such values in a bottom-up manner by distinguishing the node type of *t*, and we prove the correctness of our computation by structural induction along the way. In this correctness proof, we use additional terminology: We say that a set $\widehat{X}$ is an *extension* of *X* w.r.t. $\widehat{S}$ at *t* if it is one of those sets considered in the definition of $c_{\widehat{S},t}(X)$ that has minimum score; formally $\widehat{X}\subseteq \widehat{S}_t$, $\widehat{X}\cap \chi (t) = X$ and ${\text {score}}_{\widehat{S},t}(\widehat{X}) = c_{\widehat{S},t}(X)$. We may omit $\widehat{S}$ or *t* if they are clear from the context.

- *Leaf node*. If *t* is a leaf node, then its bag is empty and obviously $c_{\widehat{S},t}(\emptyset ) = 0$ holds.
- *Introduce node*. Let *t* be an introduce node with child $t'$, let *v* be the unique element of $\chi (t) {\setminus } \chi (t')$, let $X \subseteq \widehat{S}\cap \chi (t)$ and let $X' = X {\setminus } \{v\}$. We prove that the following equation holds:
  $$\begin{aligned} c_{\widehat{S},t}(X)= {\left\{ \begin{array}{ll} c_{\widehat{S},t'}(X')+ 1 &{}\text {if } v \in N_G[X] \cap \widehat{S}\\ c_{\widehat{S},t'}(X')- 1 &{}\text {if } v \in N_G[X] {\setminus } \widehat{S}\\ c_{\widehat{S},t'}(X')&{}\text {otherwise} \end{array}\right. } \end{aligned}$$
  First consider the case where $v \in N_G[X] \cap \widehat{S}$. Let $\widehat{X}$ be an extension of *X* at *t*, so ${\text {score}}_{\widehat{S},t}(\widehat{X})= c_{\widehat{S},t}(X)$. From $v \notin N_G[\widehat{X}{\setminus } \{v\}]_{t'}$ and $v \in N_G[\widehat{X}]_t \cap \widehat{S}$ we infer ${\text {score}}_{\widehat{S},t}(\widehat{X})= {\text {score}}_{\widehat{S},t'}(\widehat{X}{\setminus } \{v\}) + 1$. Moreover, the set $\widehat{X}{\setminus } \{v\}$ is one of the candidates considered for an extension of $X'$ in the definition of $c_{\widehat{S},t'}$, so we obtain $c_{\widehat{S},t'}(X')\le {\text {score}}_{\widehat{S},t'}(\widehat{X}{\setminus } \{v\})$. In total, this gives us $c_{\widehat{S},t}(X)\ge c_{\widehat{S},t'}(X')+ 1$. Conversely, let $\widehat{X'}$ be an extension of $X'$ at $t'$, so ${\text {score}}_{\widehat{S},t'}(\widehat{X'})= c_{\widehat{S},t'}(X')$. We distinguish two cases.
  1. If $v \in X$, then from $v \notin N_G[\widehat{X'}]_{t'}$ and $v \in N_G[\widehat{X'}\cup \{v\}]_t \cap \widehat{S}$ we infer ${\text {score}}_{\widehat{S},t}(\widehat{X'}\cup \{v\}) = {\text {score}}_{\widehat{S},t'}(\widehat{X'})+ 1$. Since $X = X' \cup \{v\}$ and $X' = \widehat{X'}\cap \chi (t')$, it holds that $X = (\widehat{X'}\cup \{v\}) \cap \chi (t)$. Hence the set $\widehat{X'}\cup \{v\}$ is one of the candidates considered for an extension of *X* in the definition of $c_{\widehat{S},t}$ and we obtain $c_{\widehat{S},t}(X)\le {\text {score}}_{\widehat{S},t}(\widehat{X'}\cup \{v\})$.
  2. Otherwise $v \notin X$. In this case $X = X'$, $v \notin \widehat{X'}$ and $X = \widehat{X'}\cap \chi (t)$. Hence the set $\widehat{X'}$ is considered in the definition of $c_{\widehat{S},t}(X)$ and we get $c_{\widehat{S},t}(X)\le {\text {score}}_{\widehat{S},t}(\widehat{X'})$. Since *v* is adjacent to an element of *X*, we infer ${\text {score}}_{\widehat{S},t}(\widehat{X'})= {\text {score}}_{\widehat{S},t'}(\widehat{X'})+ 1$.
  In both cases, we obtain $c_{\widehat{S},t}(X)\le c_{\widehat{S},t'}(X')+ 1$, so indeed $c_{\widehat{S},t}(X)= c_{\widehat{S},t'}(X')+ 1$.
  Next consider the case where $v \in N_G[X] {\setminus } \widehat{S}$. Clearly $v \notin X$. Let $\widehat{X}$ be an extension of *X* at *t*, so ${\text {score}}_{\widehat{S},t}(\widehat{X})= c_{\widehat{S},t}(X)$. From $v \notin N_G[\widehat{X}]_{t'}$ and $v \in N_G[\widehat{X}]_t {\setminus } \widehat{S}$ we now infer ${\text {score}}_{\widehat{S},t}(\widehat{X})= {\text {score}}_{\widehat{S},t'}(\widehat{X})- 1$. Similar to before, by definition of $c_{\widehat{S},t'}(X')$ we obtain $c_{\widehat{S},t'}(X')\le {\text {score}}_{\widehat{S},t'}(\widehat{X})$. In total, this gives us $c_{\widehat{S},t}(X)\ge c_{\widehat{S},t'}(X')- 1$. Conversely, let $\widehat{X'}$ be an extension of $X'$ at $t'$, so ${\text {score}}_{\widehat{S},t'}(\widehat{X'})= c_{\widehat{S},t'}(X')$. Since $v \notin \widehat{X'}$ and $X = \widehat{X'}\cap \chi (t)$, $\widehat{X'}$ is considered in the definition of $c_{\widehat{S},t}(X)$ and we get $c_{\widehat{S},t}(X)\le {\text {score}}_{\widehat{S},t}(\widehat{X'})$. Since *v* is adjacent to an element of *X*, we infer ${\text {score}}_{\widehat{S},t}(\widehat{X'})= {\text {score}}_{\widehat{S},t'}(\widehat{X'})- 1$. We obtain $c_{\widehat{S},t}(X)\le c_{\widehat{S},t'}(X')- 1$, so indeed $c_{\widehat{S},t}(X)= c_{\widehat{S},t'}(X')- 1$.
  Finally consider the remaining case where $v \notin N_G[X]$ and, in particular, $v \notin X$ holds as well as $X = X'$. Using elementary set theory with $\widehat{S}_t{\setminus } \{v\} = \widehat{S}_{t'}$ and $\chi (t) = \chi (t') \cup \{v\}$ in mind, we can prove that $\{\widehat{X}\subseteq \widehat{S}_t\mid \widehat{X}\cap \chi (t) = X\}$ is equal to $\{\widehat{X}\subseteq \widehat{S}_{t'}\mid \widehat{X}\cap \chi (t') = X'\}$. Hence a set $\widehat{X}$ is considered in the definition of $c_{\widehat{S},t}(X)$ iff it is considered in the definition of $c_{\widehat{S},t'}(X')$. For every $\widehat{X}\subseteq \widehat{S}_t$ such that $\widehat{X}\cap \chi (t) = X$, observe that $v \notin N_G[\widehat{X}]_t$, since *v* is not adjacent to any element of *X* and if it were adjacent to some element of $\widehat{X}{\setminus } X$, then $\mathcal {T}$ would not be a valid tree decomposition. This proves that every such $\widehat{X}$ has the same score at *t* and $t'$. Hence $c_{\widehat{S},t}(X)= c_{\widehat{S},t'}(X')$.
- *Forget node*. Let *t* be a forget node with child $t'$, let *v* be the unique element of $\chi (t') {\setminus } \chi (t)$ and let $X \subseteq \widehat{S}\cap \chi (t)$. We prove that the following equation holds:
  $$\begin{aligned} c_{\widehat{S},t}(X)= {\left\{ \begin{array}{ll} \min \{c_{\widehat{S},t'}(X),\; c_{\widehat{S},t'}(X \cup \{v\})\} &{} \text {if } v \in \widehat{S}\\ c_{\widehat{S},t'}(X) &{} \text {otherwise} \end{array}\right. } \end{aligned}$$
  Clearly $\widehat{S}_t= \widehat{S}_{t'}$ and all scores at forget nodes are identical to those in the respective child node. The case where $v \notin \widehat{S}$ is trivial as then $\widehat{S}\cap \chi (t) = \widehat{S}\cap \chi (t')$, i.e., the domains of $c_{\widehat{S},t}$ and $c_{\widehat{S},t'}$ are equal, and the sets considered in the definitions of $c_{\widehat{S},t}(X)$ and $c_{\widehat{S},t'}(X)$ are the same. Hence we consider the case where $v \in \widehat{S}$.
  Let $\widehat{X}$ be an extension of *X* at *t*, so $c_{\widehat{S},t}(X)= {\text {score}}_{\widehat{S},t}(\widehat{X})= {\text {score}}_{\widehat{S},t'}(\widehat{X})$. If $v \notin \widehat{X}$, then $\widehat{X}\cap \chi (t') = X$, so we obtain $c_{\widehat{S},t'}(X)\le {\text {score}}_{\widehat{S},t'}(\widehat{X})$ by definition of $c_{\widehat{S},t'}(X)$. On the other hand, if $v \in \widehat{X}$, then $\widehat{X}\cap \chi (t') = X \cup \{v\}$, so we obtain $c_{\widehat{S},t'}(X \cup \{v\}) \le {\text {score}}_{\widehat{S},t'}(\widehat{X})$. As one of these two inequalities applies, we get $c_{\widehat{S},t}(X)\ge \min \{c_{\widehat{S},t'}(X),\; c_{\widehat{S},t'}(X \cup \{v\})\}$.
  Conversely, every extension $\widehat{X'}$ of *X* at $t'$ is considered in the definition of $c_{\widehat{S},t}(X)$, so $c_{\widehat{S},t}(X)\le {\text {score}}_{\widehat{S},t}(\widehat{X'})= {\text {score}}_{\widehat{S},t'}(\widehat{X'})= c_{\widehat{S},t'}(X)$. Moreover, every extension $\widehat{X'}$ of $X \cup \{v\}$ at $t'$ is also considered in the definition of $c_{\widehat{S},t}(X)$, so $c_{\widehat{S},t}(X)\le {\text {score}}_{\widehat{S},t}(\widehat{X'})= {\text {score}}_{\widehat{S},t'}(\widehat{X'})= c_{\widehat{S},t'}(X \cup \{v\})$. If we combine these two inequalities, we get $c_{\widehat{S},t}(X)\le \min \{c_{\widehat{S},t'}(X),\; c_{\widehat{S},t'}(X \cup \{v\})\}$. Hence $c_{\widehat{S},t}(X)= \min \{c_{\widehat{S},t'}(X),\; c_{\widehat{S},t'}(X \cup \{v\})\}$.
- *Join node*. Let *t* be a join node with children $t', t''$ such that $\chi (t) = \chi (t') = \chi (t'')$, and let $X \subseteq \widehat{S}\cap \chi (t)$. We prove that the following equation holds:
  $$\begin{aligned} c_{\widehat{S},t}(X)= c_{\widehat{S},t'}(X)+ c_{\widehat{S},t''}(X)- {\text {lscore}}_{\widehat{S},t}(X)\end{aligned}$$
  Let $\widehat{X}$ be an extension of *X* at *t*, so ${\text {score}}_{\widehat{S},t}(\widehat{X})= c_{\widehat{S},t}(X)$. The set $\widehat{X'}= \widehat{X}\cap \widehat{S}_{t'}$ satisfies $\widehat{X'}\cap \chi (t') = X$, so $c_{\widehat{S},t'}(X)\le {\text {score}}_{\widehat{S},t'}(\widehat{X'})$. Symmetrically, for $\widehat{X''}= \widehat{X}\cap \widehat{S}_{t''}$ it holds that $c_{\widehat{S},t''}(X)\le {\text {score}}_{\widehat{S},t''}(\widehat{X''})$.
  There is no element of $V(G)_{t''} {\setminus } \chi (t)$ that is adjacent to an element of $\widehat{X'}{\setminus } X$, otherwise $\mathcal {T}$ would not be a valid tree decomposition. Hence $N_G[\widehat{X'}]_t = N_G[\widehat{X'}]_{t'}$, and symmetrically $N_G[\widehat{X''}]_t = N_G[\widehat{X''}]_{t''}$. This entails ${\text {score}}_{\widehat{S},t}(\widehat{X'})= {\text {score}}_{\widehat{S},t'}(\widehat{X'})$ and ${\text {score}}_{\widehat{S},t}(\widehat{X''})= {\text {score}}_{\widehat{S},t''}(\widehat{X''})$.
  Since $N_G[\widehat{X}]_t \cap \widehat{S}$ is the union of $N_G[\widehat{X'}]_t \cap \widehat{S}$ and $N_G[\widehat{X''}]_t \cap \widehat{S}$, and these latter two sets have $N_G[X] \cap \chi (t) \cap \widehat{S}$ as their intersection, we can apply the inclusion-exclusion principle to obtain ${|N_G[\widehat{X}]_t \cap \widehat{S} |} = {|N_G[\widehat{X'}]_t \cap \widehat{S} |} + {|N_G[\widehat{X''}]_t \cap \widehat{S} |} - {|N_G[X] \cap \chi (t) \cap \widehat{S} |}$. In a similar way, we get ${|N_G[\widehat{X}]_t {\setminus } \widehat{S} |} = {|N_G[\widehat{X'}]_t {\setminus } \widehat{S} |} + {|N_G[\widehat{X''}]_t {\setminus } \widehat{S} |} - {|(N_G[X] \cap \chi (t)) {\setminus } \widehat{S}) |}$. We can establish ${\text {score}}_{\widehat{S},t}(\widehat{X})= {\text {score}}_{\widehat{S},t}(\widehat{X'}) + {\text {score}}_{\widehat{S},t}(\widehat{X''})- {\text {lscore}}_{\widehat{S},t}(X)$ by putting these equations together. The inequalities we have derived before now allow us to conclude $c_{\widehat{S},t}(X)\ge c_{\widehat{S},t'}(X)+ c_{\widehat{S},t''}(X)- {\text {lscore}}_{\widehat{S},t}(X)$.
  Now let $\widehat{X'}$ and $\widehat{X''}$ be extensions of *X* at $t'$ and at $t''$, respectively. We have that $c_{\widehat{S},t'}(X)= {\text {score}}_{\widehat{S},t'}(\widehat{X'})$ and $c_{\widehat{S},t''}(X)= {\text {score}}_{\widehat{S},t''}(\widehat{X''})$. The set $\widehat{X}= \widehat{X'}\cup \widehat{X''}$ is clearly considered in the definition of $c_{\widehat{S},t}(X)$, so $c_{\widehat{S},t}(X)\le {\text {score}}_{\widehat{S},t}(\widehat{X})$. Following the same reasoning as before, we obtain ${\text {score}}_{\widehat{S},t}(\widehat{X})= {\text {score}}_{\widehat{S},t}(\widehat{X'})+ {\text {score}}_{\widehat{S},t}(\widehat{X''})- {\text {lscore}}_{\widehat{S},t}(X)$. This gives us $c_{\widehat{S},t}(X)\le c_{\widehat{S},t'}(X)+ c_{\widehat{S},t''}(X)- {\text {lscore}}_{\widehat{S},t}(X)$. Hence $c_{\widehat{S},t}(X)= c_{\widehat{S},t'}(X)+ c_{\widehat{S},t''}(X)- {\text {lscore}}_{\widehat{S},t}(X)$.

Using these recurrence relations, we can traverse the tree decomposition $\mathcal {T}$ in a bottom-up way and compute at each node *t* of $\mathcal {T}$ the value $c_{\widehat{S},t}(X)$ for each $X \subseteq \widehat{S}\cap \chi (t)$. Hence for each node of $\mathcal {T}$ we compute at most $2^w$ values, where *w* is the width of $\mathcal {T}$. By choosing the right data structure for adjacency tests [13, Exercise 7.16], each value can be computed in time $\mathcal {O}(w^3)$. Since $\mathcal {T}$ has $\mathcal {O}(w \cdot {|V(G) |})$ many nodes and $\mathcal {T}$ can be computed in fixed-parameter linear time [6], (in fact in time $2^{\mathcal {O}(w^3)} \cdot {|V(G) |}$ as observed by [9]), we thus get an algorithm with fixed-parameter linear running time for checking whether a given set $\widehat{S}$ is secure.

#### Theorem 3

Given a graph *G*, a tree decomposition of *G* of weight *w* and a set $\widehat{S}\subseteq V(G)$, we can decide in time $\mathcal {O}(2^w \cdot w^4 \cdot {|V(G) |})$ whether $\widehat{S}$ is secure in *G*.

Our algorithm can easily be adjusted to find a witness if $\widehat{S}$ is not secure, i.e., to print a subset of $\widehat{S}$ that has more attackers than defenders. After $c_{\widehat{S},t}$ has been computed for each *t*, this can be done via a final top-down traversal by a standard technique in dynamic programming on tree decompositions [26]: Alongside each value $c_{\widehat{S},t}(X)$, we store the “origin” of this value and recursively combine the origins of $c_{\widehat{S},r}(\emptyset )$, where *r* is the root of $\mathcal {T}$.

In our definition of the Secure Set Verification problem, we were only concerned with checking whether a set is secure, but we did not mention the additional constructs that we consider in this paper, like complementary vertex pairs or necessary or forbidden vertices. However, these additions pose no difficulty at all because we can just check the respective conditions in linear time.

### A Polynomial Algorithm for Secure Set on Bounded Treewidth

We now present an algorithm for finding secure sets, not just verifying whether a given set is secure. Our algorithm works by dynamic programming on a tree decomposition of the input and extends the algorithm from Sect. 4.2. For graphs of bounded treewidth, the algorithm presented in this section runs in polynomial time. However, in contrast to the algorithm in Sect. 4.2, it is not an FPT algorithm since the degree of the polynomial depends on the treewidth. This is to be expected since the problem of finding secure sets of a certain size is $\mathrm {W[1]}$-hard when parameterized by treewidth, as we have shown in Lemma 8. Our algorithm provides an upper bound for the complexity of this problem, namely membership in the class $\mathrm {XP}$.

Let *G* be a graph with a nice tree decomposition $\mathcal {T}$, and let *t* be a node of $\mathcal {T}$. In Sect. 4.2, we were given one particular secure set candidate $\widehat{S}$ that we wanted to check, so we only computed one value for each $X \subseteq \widehat{S}\cap \chi (t)$, namely the lowest score of any $\widehat{X}\subseteq \widehat{S}_t$ whose intersection with $\chi (t)$ is *X*. Here, in contrast, we cannot restrict ourselves to only one secure set candidate, and multiple candidates may have the same intersection with $\chi (t)$. We therefore compute multiple objects for each subset of $\chi (t)$, since two subsets of $V(G)_t$ that have the same intersection with $\chi (t)$ may have to be distinguished due to their subsets having different scores.

Let $S \subseteq \chi (t)$. By $F_{S}$ we denote the set of functions from $2^S$ to an integer. Let $c \in F_{S}$ and let *k* be an integer. We say that a set $\widehat{S}\subseteq V(G)_t$ is *(S, t, c, k)-characterized* if ${|\widehat{S} |} = k$, $\widehat{S}\cap \chi (t) = S$ and, for each $X \subseteq S$, it holds that $c(X) = c_{\widehat{S},t}(X)$, where $c_{\widehat{S},t}$ is the function defined in Sect. 4.2. For each $S \subseteq \chi (t)$, we now define the set

$$\begin{aligned} C_{S,t}= \{(c,k) \mid \text {there is a }(S,t,c,k)-\text {characterized set}\}. \end{aligned}$$

When *r* is the root of $\mathcal {T}$, there is a secure set of size *k* in *G* if and only if there is an element $(c,k) \in C_{\emptyset ,r}$ such that $c(\emptyset ) \ge 0$. To see this, first suppose there is a secure set $\widehat{S}$ of size *k* in *G*. Then there is a function $c: \{\emptyset \} \rightarrow \mathbb {Z}$ such that $\widehat{S}$ is $(\emptyset ,r,c,k)$-characterized, so $(c,k) \in C_{\emptyset ,r}$ and $c(\emptyset ) = c_{\widehat{S},r}(\emptyset )$, which means that $c(\emptyset )$ is the lowest score of any subset of $\widehat{S}$. Since $\widehat{S}$ is secure in *G*, this number is nonnegative. For the other direction, let $(c,k) \in C_{\emptyset ,r}$ such that $c(\emptyset ) \ge 0$. Then there is a $(\emptyset ,r,c,k)$-characterized set $\widehat{S}$, obviously of size *k*. Since $c(\emptyset ) \ge 0$, the lowest score of any subset of $\widehat{S}$ is nonnegative, which proves that $\widehat{S}$ is secure in *G*.

We now describe how to compute all such values in a bottom-up manner.

- Leaf node. If *t* is a leaf node, its bag is empty and obviously $C_{\emptyset ,t} = \{(c,0)\}$ holds, where *c* maps $\emptyset$ to 0.
- Introduce node. Let *t* be an introduce node with child $t'$ and let *v* be the unique element of $\chi (t) {\setminus } \chi (t')$. For each $S \subseteq \chi (t)$ and each function $c \in F_{S {\setminus } \{v\}}$, we define a function $c \oplus _{S,t}v :2^S \rightarrow \mathbb {Z}$. Its intended purpose is to obtain a version of *c* that applies to *t* instead of $t'$. If $v \in S$, we need to increase scores where *v* can serve as an additional defender, and otherwise we need to decrease scores where *v* can serve as an additional attacker. We now make this formal. Let $S \subseteq \chi (t)$, $X \subseteq S$, $X' = X {\setminus } \{v\}$ and $c \in F_{S {\setminus } \{v\}}$.
  $$\begin{aligned} (c \oplus _{S,t}v)(X) = {\left\{ \begin{array}{ll} c(X') + 1 &{}\text {if } v \in N_G[X] \cap S\\ c(X') - 1 &{}\text {if } v \in N_G[X] {\setminus } S\\ c(X') &{}\text {otherwise} \end{array}\right. } \end{aligned}$$
  For each $S \subseteq \chi (t)$ and each function $c \in F_{S}$ there is a unique function $c' \in F_{S {\setminus } \{v\}}$ such that $c = c' \oplus _{S,t}v$, and we denote $c'$ by ${\text {origin}}_{S,t}(c)$.
  The following statements can be proved by arguments similar to those in Sect. 4.2: Let $\widehat{S}\subseteq V(G)_{t'}$, $S = \widehat{S}\cap \chi (t')$ and $(c,k) \in C_{S,t'}$ such that $\widehat{S}$ is $(S,t',c,k)$-characterized. The set $\widehat{S}\cup \{v\}$ is $(S \cup \{v\},t,c \oplus _{S \cup \{v\},t}v,k+1)$-characterized and $\widehat{S}$ is $(S,t,c \oplus _{S,t}v,k)$-characterized. Hence $(c \oplus _{S \cup \{v\},t}v,k+1) \in C_{S \cup \{v\},t}$ and $(c \oplus _{S,t}v,k) \in C_{S,t}$. Conversely, let $\widehat{S}\subseteq V(G)_{t}$, $S = \widehat{S}\cap \chi (t)$ and $(c,k) \in C_{S,t}$ such that $\widehat{S}$ is (*S*, *t*, *c*, *k*)-characterized, and let $c' = {\text {origin}}_{S,t}(c)$ and $k' = k - {|S \cap \{v\} |}$. The set $\widehat{S}{\setminus } \{v\}$ is $(S {\setminus } \{v\},t',c',k')$-characterized. Hence $(c',k') \in C_{S {\setminus } \{v\},t'}$.
  From these observations, the following equation follows for every $S \subseteq \chi (t)$:
  $$\begin{aligned} C_{S,t}= \{(c \oplus _{S,t}v, k+{|S \cap \{v\} |}) \mid (c,k) \in C_{S {\setminus } \{v\},t'}\} \end{aligned}$$
- Forget node. Let *t* be a forget node with child $t'$ and let *v* be the unique element of $\chi (t') {\setminus } \chi (t)$. For each $S \subseteq \chi (t)$ and each function $c \in F_{S \cup \{v\}}$, we define a function $c \ominus _{S,t}^{\in }v$, and for each $S \subseteq \chi (t)$ and each function $c \in F_{S}$, we define a function $c \ominus _{S,t}^{\notin }v$. Each of these functions maps every subset of *S* to an integer.
  $$\begin{aligned} \left( c \ominus _{S,t}^{\in }v\right) (X)= & {} \min \{c(X),\; c(X \cup \{v\})\}\\ \left( c \ominus _{S,t}^{\notin }v\right) (X)= & {} c(X) \end{aligned}$$
  Next we define functions ${\text {origin}}_{S,t}^{\in }$ and ${\text {origin}}_{S,t}^{\notin }$ that map each element of $F_{S}$ to a set of elements of $F_{S \cup \{v\}}$ and $F_{S}$, respectively:
  $$\begin{aligned} {\text {origin}}_{S,t}^{\in }(c)= & {} \left\{ c' \in F_{S \cup \{v\}}\mid c = c' \ominus _{S,t}^{\in }v\right\} \\ {\text {origin}}_{S,t}^{\notin }(c)= & {} \left\{ c' \in F_{S}\mid c = c' \ominus _{S,t}^{\notin }v\right\} \end{aligned}$$
  The following statements can be proved by arguments similar to those in Sect. 4.2: Let $\widehat{S}\subseteq V(G)_{t'}$, $S = \widehat{S}\cap \chi (t')$ and $(c,k) \in C_{S,t'}$ such that $\widehat{S}$ is $(S,t',c,k)$-characterized. If $v \in \widehat{S}$, then $\widehat{S}$ is $(S {\setminus } \{v\},t,c \ominus _{S,t}^{\in }v,k)$-characterized and $(c \ominus _{S,t}^{\in }v,k) \in C_{S {\setminus } \{v\},t}$; otherwise $\widehat{S}$ is $(S,t,c \ominus _{S,t}^{\notin }v,k)$-characterized and $(c \ominus _{S,t}^{\notin }v,k) \in C_{S,t}$. Conversely, let $\widehat{S}\subseteq V(G)_{t}$, $S = \widehat{S}\cap \chi (t)$ and $(c,k) \in C_{S,t}$ such that $\widehat{S}$ is (*S*, *t*, *c*, *k*)-characterized. If $v \in \widehat{S}$, then there is some $c' \in {\text {origin}}_{S,t}^{\in }(c)$ such that $\widehat{S}$ is $(S \cup \{v\},t',c',k)$-characterized and $(c',k) \in C_{S \cup \{v\},t'}$; otherwise there is some $c' \in {\text {origin}}_{S,t}^{\notin }(c)$ such that $\widehat{S}$ is $(S,t',c',k)$-characterized and $(c',k) \in C_{S,t'}$.
  From these observations, the following equation follows for every $S \subseteq \chi (t)$:
  $$\begin{aligned} C_{S,t}= \left\{ \left( c \ominus _{S,t}^{\in }v,k\right) \mid (c,k) \in C_{S \cup \{v\},t'}\right\} \cup \left\{ \left( c \ominus _{S,t}^{\notin }v,k\right) \mid (c,k) \in C_{S,t'}\right\} \end{aligned}$$
- Join node. Let *t* be a join node with children $t', t''$ such that $\chi (t) = \chi (t') = \chi (t'')$. For each $S \subseteq \chi (t)$, and each $c', c'' \in F_{S}$, we define a function $c' \otimes _{S,t}c''$, which maps each subset of *S* to an integer.
  $$\begin{aligned} (c' \otimes _{S,t}c'')(X) = c'(X) + c''(X) - {\text {lscore}}_{S,t}(X)\end{aligned}$$
  Next we define a function ${\text {origin}}_{S,t}$ that maps each element of $F_{S}$ to a subset of $F_{S}\times F_{S}$:
  $$\begin{aligned} {\text {origin}}_{S,t}(c)= \{(c',c'') \in F_{S}\times F_{S}\mid c = c' \otimes _{S,t}c''\} \end{aligned}$$
  The following statements can be proved by arguments similar to those in Sect. 4.2: Let $\widehat{S'}\subseteq V(G)_{t'}$, $\widehat{S''}\subseteq V(G)_{t''}$, $S = \widehat{S'}\cap \widehat{S''}$, $(c',k') \in C_{S,t'}$ and $(c'',k'') \in C_{S,t''}$ such that $\widehat{S'}$ is $(S,t',c',k')$-characterized and $\widehat{S''}$ is $(S,t'',c'',k'')$-characterized, and let $c = c' \otimes _{S,t}c''$ and $k = k' + k'' - {|S |}$. The set $\widehat{S'}\cup \widehat{S''}$ is (*S*, *t*, *c*, *k*)-characterized and $(c,k) \in C_{S,t}$. Conversely, let $\widehat{S}\subseteq V(G)_{t}$, $S = \widehat{S}\cap \chi (t)$ and $(c,k) \in C_{S,t}$ such that $\widehat{S}$ is (*S*, *t*, *c*, *k*)-characterized. There is some $(c',c'') \in {\text {origin}}_{S,t}(c)$ as well as integers $k', k''$ such that $k = k' + k'' - {|S |}$, the set $\widehat{S}\cap V(G)_{t'}$ is $(S,t',c',k')$-characterized and $\widehat{S}\cap V(G)_{t''}$ is $(S,t'',c'',k'')$-characterized. Hence $(c',k') \in C_{S,t'}$ and $(c'',k'') \in C_{S,t''}$.
  From these observations, the following equation follows for every $S \subseteq \chi (t)$:
  $$\begin{aligned} C_{S,t}= \{(c' \otimes _{S,t}c'', k' + k'' - {|S |}) \mid (c',k') \in C_{S,t'},\; (c'',k'') \in C_{S,t''}\} \end{aligned}$$

We can now traverse the tree decomposition $\mathcal {T}$ in a bottom-up way and at each node *t* of $\mathcal {T}$ compute the set $C_{S,t}$ for each $S \subseteq \chi (t)$. Let *n* denote the number of vertices of *G* and *w* denote the width of $\mathcal {T}$. Every element of $C_{S,t}$ is a pair (*c*, *k*), where *c* is a function that maps each subset of *S* to an integer between $-n$ and *n*, there are at most $2^w$ subsets of *S*, and *k* is an integer between 0 and *n*. Hence there are at most $(2n+1)^{2^w} \cdot (n+1)$ elements of $C_{S,t}$. Each individual element of $C_{S,t}$ can be computed in time $\mathcal {O}(2^w)$. Finally, there are at most $2^w$ possible values for *S* and $\mathcal {O}(wn)$ many nodes in $\mathcal {T}$. We thus get an algorithm that takes as input an integer *k* together with a graph *G* whose treewidth we denote by *w*, and determines in time $f(w) \cdot n^{g(w)}$ whether *G* admits a secure set of size *k*, where *f* and *g* are functions that only depend on *w*.

This algorithm for Exact Secure Set obviously also gives us an algorithm for Secure Set by checking all solution sizes from 1 to *k*. Finally, we can easily extend it to accommodate complementary vertex pairs as well as necessary and forbidden vertices. Hence we get the following $\mathrm {XP}$ membership result:

#### Theorem 4

Secure Set, Exact Secure Set, ${\textsc {Secure}\,\textsc {Set}^\textsc {F}}$, ${\textsc {Exact}\,\textsc {Secure}\,\textsc {Set}^\textsc {F}}$, ${\textsc {Secure}\,\textsc {Set}^\textsc {FN}}$, ${\textsc {Exact}\,\textsc {Secure}\,\textsc {Set}^\textsc {FN}}$, ${\textsc {Secure}\,\textsc {Set}^\textsc {FNC}}$ and ${\textsc {Exact}\,\textsc {Secure}\,\textsc {Set}^\textsc {FNC}}$ can be solved in polynomial time if the treewidth of the input is bounded by a constant.

By keeping track of the origins of our computed values during our bottom-up traversal of the tree decomposition, we can easily adapt the algorithm to find solutions if they exist.

## Conclusion

In this work, we have solved a complexity problem in graph theory that, to the best of our knowledge, has remained open since the introduction of secure sets [11] in 2007. We have shown that the problem of deciding whether, for a given graph *G* and integer *k*, *G* possesses a non-empty secure set of size at most *k* is $\mathrm {\Sigma ^P_2}$-complete. We moreover obtained $\mathrm {\Sigma ^P_2}$-completeness for seven further variants of this problem.

In the second part of this paper, we analyzed the complexity of the Secure Set problem parameterized by the treewidth of the input graph. In particular, we showed that bounded treewidth does not make the problem fixed-parameter tractable unless $\mathrm {FPT}= \mathrm {W[1]}$. Nevertheless, we provided a polynomial-time algorithm for finding secure sets on graphs of bounded treewidth and thus showed membership in the class $\mathrm {XP}$. As a positive result, we could show that the $\mathrm {\text {co-}NP}$-complete problem of verifying whether a given set is secure can be solved in fixed-parameter linear time when parameterized by treewidth.

There are several interesting directions for future research. One open question is which additional restrictions beside bounded treewidth need to be imposed on Secure Set instances to achieve fixed-parameter tractability. On the other hand, the Secure Set Verification problem may remain FPT for parameters that are less restrictive than treewidth. We showed $\mathrm {W[1]}$-hardness and $\mathrm {XP}$-membership of Secure Set, so a tight bound is still lacking, albeit perhaps more of theoretical interest due to the fact that problems at a certain level of the weft hierarchy generally do not admit faster algorithms than problems at higher levels. To classify a problem as FPT w.r.t. treewidth, a common approach is to express it in monadic second-order logic (MSO) and then invoke Courcelle’s Theorem [12], which immediately proves that the problem is FPT. We showed that Secure Set Verification is FPT, but it is not clear if it can be expressed in MSO. If it cannot, then our FPT result could hint at possible extensions of MSO whose model-checking problem is still FPT. Similarly, we believe that MSO can be extended in such a way that Secure Set can be expressed and that a variant of Courcelle’s Theorem for showing membership in $\mathrm {XP}$ instead of $\mathrm {FPT}$ holds. Finally, some of our results seem to be transferable to (variants of) the Defensive Alliance problem, so it would be interesting to investigate if some of our reductions and algorithms can help in the study of such related problems.

## Footnotes

1 To be precise, [22] shows that a slight variant of Secure Set is tractable on trees, since Secure Set on trees is trivial. Our results, however, also imply $\mathrm {W[1]}$-hardness for this particular variant.
